# Reduced *Vrk2* expression is associated with higher risk of depression in humans and mediates depressive-like behaviors in mice

**DOI:** 10.1186/s12916-023-02945-0

**Published:** 2023-07-14

**Authors:** Mei-Yu Yin, Lei Guo, Li-Juan Zhao, Chen Zhang, Wei-Peng Liu, Chu-Yi Zhang, Jin-Hua Huo, Lu Wang, Shi-Wu Li, Chang-Bo Zheng, Xiao Xiao, Ming Li, Chuang Wang, Hong Chang

**Affiliations:** 1 Key Laboratory of Animal Models and Human Disease Mechanisms of the Chinese Academy of Sciences and Yunnan Province, Kunming Institute of Zoology, Chinese Academy of Sciences, Kunming, Yunnan China; 2 Kunming College of Life Science, University of Chinese Academy of Sciences, Kunming, Yunnan China; 3 Zhejiang Key Laboratory of Pathophysiology, Health Science Center, Ningbo University, Ningbo, Zhejiang China; 4 School of Basic Medical Science, Health Science Center, Ningbo University, Ningbo, Zhejiang China; 5 Clinical Research Center & Division of Mood Disorders, Shanghai Mental Health Center, Shanghai Jiao Tong University School of Medicine, Shanghai, China; 6 Shanghai Key Laboratory of Psychotic Disorders, Shanghai, China; 7 School of Pharmaceutical Science and Yunnan Key Laboratory of Pharmacology for Natural Products, Kunming Medical University, Kunming, Yunnan China

**Keywords:** Major depression, *VRK2*, Lower expression, Behavioral abnormalities, Dendritic spines

## Abstract

**Background:**

Genome-wide association studies (GWAS) have reported single-nucleotide polymorphisms (SNPs) in the VRK serine/threonine kinase 2 gene (*VRK2*) showing genome-wide significant associations with major depression, but the regulation effect of the risk SNPs on *VRK2* as well as their roles in the illness are yet to be elucidated.

**Methods:**

Based on the summary statistics of major depression GWAS, we conducted population genetic analyses, epigenome bioinformatics analyses, dual luciferase reporter assays, and expression quantitative trait loci (eQTL) analyses to identify the functional SNPs regulating *VRK2*; we also carried out behavioral assessments, dendritic spine morphological analyses, and phosphorylated 4D-label-free quantitative proteomics analyses in mice with *Vrk2* repression.

**Results:**

We identified a SNP rs2678907 located in the 5’ upstream of *VRK2* gene exhibiting large spatial overlap with enhancer regulatory marks in human neural cells and brain tissues. Using luciferase reporter gene assays and eQTL analyses, the depression risk allele of rs2678907 decreased enhancer activities and predicted lower *VRK2* mRNA expression, which is consistent with the observations of reduced *VRK2* level in the patients with major depression compared with controls. Notably, *Vrk2*^−/−^ mice exhibited depressive-like behaviors compared to *Vrk2*^+/+^ mice and specifically repressing *Vrk2* in the ventral hippocampus using adeno-associated virus (AAV) lead to consistent and even stronger depressive-like behaviors in mice. Compared with *Vrk2*^+/+^ mice, the density of mushroom and thin spines in the ventral hippocampus was significantly altered in *Vrk2*^−/−^ mice, which is in line with the phosphoproteomic analyses showing dysregulated synapse-associated proteins and pathways in *Vrk2*^−/−^ mice.

**Conclusions:**

*Vrk2* deficiency mice showed behavioral abnormalities that mimic human depressive phenotypes, which may serve as a useful murine model for studying the pathophysiology of depression.

**Supplementary Information:**

The online version contains supplementary material available at 10.1186/s12916-023-02945-0.

## Background

Major depression is a polygenic illness with considerable heritability [1], and multiple genome-wide association studies (GWAS) have been carried out to identify risk genes [2–4]. Owing to the effects of linkage disequilibrium (LD), both causal variants (with direct effects) and their LD-associated variants (with indirect effects) in a GWAS locus will be highlighted, and identifying the causal variants is crucial for understanding the biological basis and pathological mechanism of depression [5]. Several approaches have been applied to refine the most credible causal variants, such as whether they regulate promoter or enhancer activities by affecting the binding affinities to transcription factors or enhancer complex. Meanwhile, there is also emerging evidence that cross-populations fine-mapping resolution based on LD could help define the causal and LD-associated variants. Indeed, despite large differences in LD structures, it is widely acknowledged that causal variants have usually shown consistent effects across populations, whereas LD-associated variants may have inconsistent effects [6].

In the depression GWAS using 23andMe sample of European ancestry, single-nucleotide polymorphism (SNP) rs1518395 in the VRK serine/threonine kinase 2 (*VRK2*) gene exhibited genome-wide significant association (*P* = 4.32 ⨯ 10^–12^) [2] and interacted with environmental pollution to affect the development of depression [7], but this SNP showed no evidence of association in the Han Chinese depression GWAS (*P* = 0.791) [8]. Later, in the European depression GWAS including a combination of 23andMe, UK Biobank, and PGC2 samples [3], new risk SNPs spanning *VRK2* were discovered (*P* < 5.00 ⨯ 10^–8^). These newly identified SNPs were in low LD with rs1518395 and showed nominal associations with depression in the Han Chinese GWAS (*P* < 0.05) [8]. *VRK2* encodes a transmembrane serine/threonine kinase that participates in multiple cellular functions [9–11] and is expressed in both neurons and microglia [12, 13]. Previous studies have reported that deletion of *Vrk2* in zebrafish causes aggressive behaviors and alters social preference [14]. Whereas, *Vrk2*-deficient mice showed defects in social behaviors, contextual fear memory, and spatial memory [13]. However, the depressive-like behaviors which might be caused by *Vrk2* were not examined.

We speculated that causal variants within the *VRK2* gene may show consistent effects on the risk of depression across distinct populations. An integrative approach combining cross-populations replication and epigenome bioinformatics analyses, followed by functional assays, may facilitate the identification of such causal variants. Indeed, we identified a functional SNP rs2678907, in which the depression risk allele predicted decreased DNA enhancer activities and lower *VRK2* mRNA expression. Further in vivo murine experiments demonstrated that repression of *Vrk2* in the ventral hippocampus leads to depressive-like behaviors, as well as abnormalities in the dendritic spine morphogenesis and dysregulation of synapse-associated proteins/processes.

## Methods

### Descriptions about depression GWAS sample of European ancestry

In 23andMe sample (75,607 cases and 231,747 controls) [2], the definition of depression was based on responses to web-based surveys, in which case individuals were required to self-report as having received a clinical diagnosis or treatment for depression. Due to restrictions on data sharing policy, the 23andMe GWAS did not publicly release genome-wide summary statistics but only provided the summary statistics of SNPs spanning the *VRK2* region (chr2:57917222–58482646 (hg19)) in the Supplementary Materials of their GWAS [2].

In UK Biobank sample (127,552 cases and 233,763 controls) [15], broad definition of depression was utilized, in which cases were required to answer “yes” to the questions “Have you ever seen a general practitioner for nerves, anxiety, tension or depression?” or “Have you ever seen a psychiatrist for nerves, anxiety, tension or depression?” Individuals with self-reported bipolar disorder, schizophrenia, or personality disorder were excluded.

In PGC2 samples (43,204 cases and 95,680 controls) [4], a clinical diagnosis of lifetime major depressive disorder was applied to define cases, and most controls randomly selected from local populations were screened for the absence of lifetime major depressive disorder.

GWAS meta-analysis summary statistics of UK Biobank and PGC2 samples were publicly released [3]. We used PLINK v1.9 to combine the summary statistics (e.g., effect allele, odds ratio (OR), standard error (SE), *P* value) of the SNPs spanning the *VRK2* region (chr2:57917222–58482646 (hg19)) across 23andMe, UK Biobank, and PGC2 samples and conduct the meta-analysis [16].

### Depression case–control sample of Han Chinese origin

The CONVERGE Consortium has conducted a depression GWAS in Han Chinese individuals, which included 5303 cases and 5337 non-psychiatric controls [8]. As described in the original study, cases were diagnosed using the Composite International Diagnostic Interview (WHO lifetime version 2.1; Chinese version) according to the DSM-IV criteria. Controls were recruited either from local communities or from the patients who underwent minor surgical procedures at the general hospitals.

### Linkage disequilibrium (LD) analysis and functional prediction

The regional association results of *VRK2* SNPs were plotted using LocusZoom [17]. Haploview (version 4.1) [18] was used to examine LD between SNPs using the *r*^2^ algorithm based on the SNP data from the 1000 Genomes Project [19]. We applied ENCODE [20] and HaploReg v4.2 [21] for SNP functional prediction analyses, which is primarily based on open-chromatin peaks defined by DNase I hypersensitivity and ChIP-Seq peaks of histone modifications such as H3K4me1 in brain hippocampal tissues and multiple types of cells (SK-N-SH, bipolar neurons originated from GM23338, neural progenitor cells differentiated from H9). Detailed information about the assays and methods in ENCODE project can be found online. We also used a web-based tool GWAVA (genome-wide annotation of variants), which aims to prioritize regulatory elements according to evolutionary conservation and GC-content as well as experimentally epigenetic features in ENCODE/GENCODE, to further predict whether the functional elements overlapped with the tested SNPs [22].

### Cloning the DNA fragments containing rs2678907 and dual luciferase reporter gene assays

Dual luciferase reporter gene assays were employed to analyze if the DNA fragments containing either G or A allele at the rs2678907 site show different enhancer activity. DNA sequences covering rs2678907 (Chr2:58133522) were amplified encompassing Chr2:58132622–58134828 (hg19). Site-directed mutagenesis was employed to obtain either the G or A allele at the rs2678907 site, which were then constructed into the pGL3-promoter plasmids. The pGL3-promoter plasmid with DNA fragment containing either G-allele or A-allele in equal amounts was transiently co-transfected into human SK-N-SH, U251, and HEK293T cell lines with pRL-TK plasmid using Lipofectamine 3000 transfection reagent (Invitrogen). Thirty-six hours post-transfection, cells were collected, and luciferase activity were measured by Dual-Luciferase Reporter Assay System (Promega). All assays were performed with minimum of four replications in at least three independent experiments. GraphPad Prism 9 was used for the statistical analysis, and two-tailed *t*-test was used to compare the two groups. All experimental data are presented as mean ± standard deviation (SD).

### Datasets for expression quantitative trait loci (eQTL)

The eQTL results in the CommonMind datasets (209 schizophrenia patients, 52 affective disorder cases, and 206 controls) were examined [23]. In brief, the gene expression was measured using polyA^+^ RNA-sequencing, followed by normalization via adjusting for 20 surrogate variables (SVs), RNA integrity number (RIN), RIN2, diagnosis, sex, institution, PMI, clustered LIB, and age of disease onset. The eQTL analysis was performed according to the formula: adjusted gene expression ~ SNP + ancestry vectors + diagnosis.

We also obtained RiboZero RNA-seq eQTL results in the postmortem caudate nucleus tissues of 444 individuals (154 schizophrenia patients, 44 bipolar patients, and 246 controls) from Lieber Institute for Brain Development [24]. The eQTL analyses were performed using Matrix eQTL with log_2_ transformed RPKM adjusted for diagnosis, sex, SNP PCs, and expression PCs.

A third Braineac eQTL dataset comprising of independent individuals of European ancestry was also utilized [25]. In this dataset, the UK Brain Expression Consortium (UKBEC) has generated genotype (using Illumina Omni1-quad and Immunochip arrays) and expression data (using Affymetrix Human Exon 1.0 ST arrays) for brain tissues from 134 control individuals of European descent, and we retrieved the eQTL results in the frontal cortex for the present study. As described in the original study, the eQTL analysis was conducted using Matrix eQTL for each normalized expression profile against SNP.

### Differential expression analyses

Jansen et al. [26] previously examined mRNA expression in the peripheral venous blood of remitted depression (present in lifetime but not at the current interview, *n* = 635) or current depression (within the 6 months prior to interview, *n* = 882) and control subjects with no lifetime history of depression or anxiety disorders (*n* = 331), using Affymetrix U219 arrays and the GeneTitan System. For differential expression analysis between depression patients and controls, *P* value and effect size were computed for the associations between depression and gene expression based on a linear model consisting of empirical covariates.

#### Animals

Wild-type C57BL/6 J male mice (5–6 weeks old) were purchased from GemPharmatech Co., Ltd. (Nanjing, China) and bred for 2–3 weeks to perform experiments. *Vrk2*-knockout (KO) mice (*Vrk2*^−/−^, Stock No. T013808, C57BL/6 J background) were ordered from GemPharmatech Co., Ltd. (Nanjing, China). Exon 3 to exon 7 of *Vrk2* were deleted by CRISPR/Cas9 to create the knockout mouse model. The mice were housed in a 12-h light/dark room (lights on at 08:00, lights off at 20:00) with controlled temperature (23 ± 2 °C) as well as 50–60% relative humidity and free to access to food and water. All animal experiments were carried out according to the guidelines (developed by the National Advisory Committee for Laboratory Animal Research) for ethical conduct in the care and use of animals, and all protocols were approved by the Animal Ethic Committee of Kunming Institute of Zoology (NO: SMKX-2021–01-001).

### *VRK2*-shRNA and overexpression plasmid construction, adeno-associated virus (AAV) package and purification

Knockdown of mouse *Vrk2* was achieved by short hairpin RNA (shRNA). Three shRNA sequences targeting *Vrk2* were designed: 5’-GATGCAAGACATGTCATAA-3’ (shRNA1), 5’-CCGTCCTACAACTTGGCAT-3’ (shRNA4), and 5’-GGGCCATAATGGGACAATA-3’ (shRNA5), and a non-specific target sequence (5’-GATTTGCTGTTCGCCCAAG-3’) on mouse genome was employed as negative control. The shRNA sequences were constructed into pAAV-CAG-tdTomato (Addgene, #59,462). To overexpress Vrk2, the full-length murine *Vrk2* coding sequence with the C-terminal Flag-tag was constructed into the pAAV-CAG-tdTomato vector (Addgene, #59,462), and the empty vector plasmid was utilized as the control. Sanger sequencing was used to ensure the correct construction.

Recombinant plasmids were co-transfected with packaging plasmids (AAV-DJ and pHelper) into HEK293T cells by linear polyethylenimine 40 kDa (Polysciences) [27]. Seventy-two hours post-transfection, cells were collected, resuspended (0.15 M NaCl; 20 mM Tris–HCl) and subjected to 4 freeze–thaw cycles in liquid nitrogen and 37 °C water bath to release AAV virus. Benzonase treatment (Sigma, #E1014) was used to remove extraviral DNA contamination at 37 °C for 30 min and the reaction mixture was centrifuged at 8000 × *g* for 90 min at 4 °C. The supernatant containing AAV virus was collected and then added to 15%, 25%, 40%, 54% iodixanol (Sigma-Aldrich) step gradient and centrifuged at 60,000 × *g* for 3 h at 4 °C. Finally, 100–200 μL AAV virus were purified by Amicon ultrafiltration tube (EMD Millipore).

### Stereotactic injection

Wild-type C57BL/6 J male mice and *Vrk2*-knockout (KO) mice received stereotaxic injection at 7–8 weeks of age. The mice were fixed on the brain stereotactic apparatus horizontally (RWD Life Science Co., Ltd., Shenzhen, China) and anesthetized with isoflurane gas. A longitudinal incision was made to expose the skull surface. Set the anterior fontanelle as the origin and drill holes according to the ventral hippocampal coordinates (AP – 3.08 mm; ML ± 3.20 mm; DV − 4.0 mm/ − 3.5 mm) with a skull drill (RWD Life Science Co., Ltd., Shenzhen, China). A micro-syringe (RWD Life Science Co., Ltd., Shenzhen, China) was positioned above the drilled hole according to the coordinates and was used to take in the AAV virus (titer = 5 × 10^12^ IU/mL), then the injection needle was slowly lowered through the hole into the target brain area. Five minutes later, a syringe pump controller was used (RWD Life Science Co., Ltd., Shenzhen, China) to perform a bilateral injection of 1 µl per side at a speed of 2 nL/s. Ten minutes after injection, the needle was slowly withdrawn to prevent the virus from leaking out. The scalp was sutured and the mice were then returned to the cage after awakening.

### Real-time quantitative PCR

Total RNAs from fresh hippocampal tissues were extracted with TRIzol Reagent (Life Technologies). Complementary DNA (cDNA) was synthesized from 1 μg of RNA using the RevertAid First Strand cDNA Synthesis Kit (Thermo Scientific). The mRNA expression of *Vrk2* was quantified through real-time quantitative PCR (RT-qPCR) by using FastStart Universal SYBR Green Master (Roche) and the reaction was performed on the CFX96 real-time PCR system (Bio-Rad, USA). *Gapdh* was used as an internal control and the relative gene expression values were presented as the means of 2^^–ΔΔCt^. Primer sequences for RT-qPCR are as follows: *Gapdh* (Forward: 5’-ATGGTGAAGGTCGGTGTGAA-3’, Reverse: 5’-CAATCTCCACTTTGCCACTGC-3’) and *Vrk2* (Forward: 5’- GTTCATGGTGATATAAAAGCCGCA-3’, Reverse: 5’-TGGCCCTTTCTGGGATCTTC-3’).

### Western blot

The hippocampus tissues were homogenized by a tissue homogenizer in RIPA lysate with protease and phosphatase inhibitors. The mixture was centrifuged and the supernatant was subjected to protein quantification using a BCA protein assay kit (Thermo Scientific, USA). After being denatured at 95 °C with a fivefold loading buffer, protein samples were separated on a 10% SDS-PAGE gel and transferred to polyvinylidene difluoride (PVDF) membranes (EMD Millipore, USA). The transferred membranes were blocked with 5% BSA and then incubated with the primary antibody overnight at 4 °C. After that, the secondary antibody was incubated for 60 min at room temperature with a peroxidase-conjugated secondary antibody. Finally, the blots were reacted with the Tanon ECL Chemiluminescent reagent (#180-5001W) and the images were captured by Tanon 5200 imaging system. The primary antibodies were as follows: anti-Vrk2 (#12946–1-AP, Proteintech, USA) and anti-Gapdh (#60004–1-Ig, Proteintech, USA).

### Open field test (OFT)

The open-field box (40 cm L × 40 cm W × 40 cm H) floor was virtually divided into 9 equal squares, with the central square considered as the central area and the other 8 outer squares designated as the peripheral area. Each mouse was placed in the same corner of the open field box, facing the wall, and permitted to explore the environment freely for 5 min. Their activity tracks were recorded by a video camera. The time and distance in the central area were analyzed by SuperMaze software (RWD Life Science Co., Ltd., Shenzhen, China) to evaluate their anxiety. At the end of each experiment, the open-field box was cleaned with 75% alcohol.

The murine behavioral data were analyzed using GraphPad Prism software and were presented as mean ± SD. For normally distributed data evaluated by Shapiro–Wilk test, two-tailed *t*-test was used to assess the difference between two groups, and one-way analysis of variance (ANOVA) was performed for more than two groups. For all the behavioral analyses, we only used male mice, as according to previous studies, female mice are more susceptible to individual variances brought by the estrous cycle, which might lead to unreliable results [28].

### Elevated plus maze (EPM)

The elevated plus maze (EPM) is composed of a pair of open arms (33 cm L × 8 cm W) and a pair of closed arms (30 cm L × 6 cm W). The mice were put into the central area facing one of the open arms. SuperMaze software (RWD Life Science Co., Ltd., Shenzhen, China) was used to record the number and time of entering the open arm and the closed arm within 6 min [29, 30]. Their activity tracks were recorded by a video camera. The percentage of time and distance that the mice entered the open arm were analyzed. At the end of each experiment, the elevated plus maze was cleaned with 75% alcohol.

### Rotarod test

The general motor function was evaluated on the rotarod test. Mice were placed on the rotarod (RWD Life Science Co., Ltd., Shenzhen, China) and trained to walk on the rotarod at a constant speed of 10 rpm for 5 min. After 2 days of training, the motor capacity of mice was tested using the accelerating task (from 4 to 40 rpm in 5 min). The experiment ended when the mice fell or gripped and spun around for one complete revolution. Each mouse underwent three trails with an intertrial interval of 60 min. The average latency time to fall for the three trials was used as a statistical indicator.

### Tail suspension test (TST)

TST was used to detect desperation behavior in mice [31]. The mouse tail tip was taped about 1 cm on the hook of the suspension tail test chamber (20 cm L × 20 cm W × 32 cm H). The time of each activity state was recorded using SuperTST software (RWD Life Science Co., Ltd., Shenzhen, China). According to previous studies [32] and the analytical software, the behavior states of mice were categorized according to activity levels: (1) immobility: the mouse was suspended without any activity behavior, (2) swinging: when a mouse repeatedly moved its paw or significantly moved its body in a vertical position, and (3) curling: the mice’s bodies make active twisting movements. The experiment process for a total of 6 min, and the time duration of each behavior state in the last 4 min was analyzed.

### Sucrose preference test (SPT)

SPT is used to detect the absence of pleasure in experimental animals, which is one of the main indicators of depression [33]. First, the mice are housed in a single cage, and two water bottles, one with clean water and the other with 1% sucrose solution, are placed on the cage and acclimated for 48 h, with the position of the two water bottles changing every 24 h. Mice were deprived of drinking water for 24 h at the end of the acclimatization period. During the test, the water bottle containing clean water and the 1% sucrose solution remained on the mouse cage, and the test lasted a total of 12 h, with the positions of the two water bottles changing every 6 h, and the amount of clean water and the 1% sucrose solution consumed was recorded. Sucrose preference % = 1% sucrose solution consumption / (1% sucrose solution consumption + clean water consumption) × 100%.

### Neuronal sparse labeling

Individual neurons’ local dendritic morphology was recreated by sparse and highly visible tagging of neurons with fluorescent proteins carried by adenovirus (Brain Case, #BC-SL001) to precisely clarify the quantity and form of their dendritic spines. By stereotaxic brain injection, the adenovirus was administered to the ventral hippocampus (AP – 3.08 mm; ML ± 3.20 mm; DV − 3.8 mm) of 7–8-week-old mice (*Vrk2*^+/+^ and *Vrk2*^−/−^) in a volume of 200 nL on each side. Four weeks after surgery, mice were perfused with PBS and 4% paraformaldehyde, and undamaged brain tissue was extracted and fixed overnight in 4% paraformaldehyde at 4 °C, followed by dehydration in 30% sucrose solution at 4 °C. The dehydrated brain tissues were embedded with OCT and placed on a frozen section machine to create 50-μm coronal brain slices. The slices were sealed with anti-quenching sealer and imaged using a laser confocal microscope at high magnification. Secondary or tertiary dendrites in the hippocampal pyramidal neurons were used as photographic objects, and statistical analysis was carried out using NeuronStudio [34] and Image-J software. Dendritic spines can be divided into mushroom-shaped spines (head-neck ratio ≥ 1.5), thin and long spines (head-neck ratio < 1.5, length/head diameter ≥ 2), and short and thick spines (head–neck ratio < 1.5, length/head diameter < 2), and the number of spines in a dendrite of 10 μm length was used as its density, and the density of different morphological dendritic spines was analyzed and counted. Before statistical analysis, the data were evaluated whether they were normally distributed by Shapiro–Wilk test, and two-tailed *t*-test was used to compare the total number of dendritic spines, and the two-way ANOVA was used to determine the various morphologies. All experimental data are presented as mean ± SD.

### Phosphoproteomic analysis

Hippocampus tissues (*n* = 3, each group, *Vrk2*^+/+^ and *Vrk2*^−/−^) were isolated and the homogenized tissues were subjected to protein extraction by SDT (4% SDS, 100 mM Tris–HCl, 1 mM DTT, pH 7.6) buffer. The protein concentrations were determined by the BCA (Bio-Rad, USA) method. A suitable amount of protein from each sample was trypsinized using the filter-aided proteome preparation (FASP) method [35], and the peptides were desalted using a C18 Cartridge, vacuum centrifuged, and dissolved in 40 l of 0.1% formic acid solution. The peptide content was determined using UV spectroscopy at 280 nm. Each peptide solution was vacuum-lyophilized and then enriched with the High-SelectTM Fe-NTA Phosphopeptides Enrichment Kit (Thermo Scientific). The enriched phosphorylated peptides were concentrated under vacuum and diluted in 20 L of 0.1% formic acid solution before being analyzed by mass spectrometry. The LC–MS/MS analysis was carried out using a Bruker TimsTOF Pro mass spectrometer. The MS raw data for each sample were combined and searched using the MaxQuant 1.6.14 software for identification and quantitation analysis. The differentially phosphorylated proteins were selected on the basis of the combination of |log_2_(fold change)|≥ 1 and *P* ≤ 0.05. The differentially phosphorylated proteins then underwent enrichment analyses of Kyoto Encyclopedia of Genes and Genomes (KEGG), Gene Ontology (GO), Reactome pathway [36], and Monarch Human Phenotype Ontology (HPO) [37] using the STRING dataset (version 11.5) [38].

## Results

### The strategy and logic of choosing VRK2 SNPs for genetic analyses

**Fig. 1:**
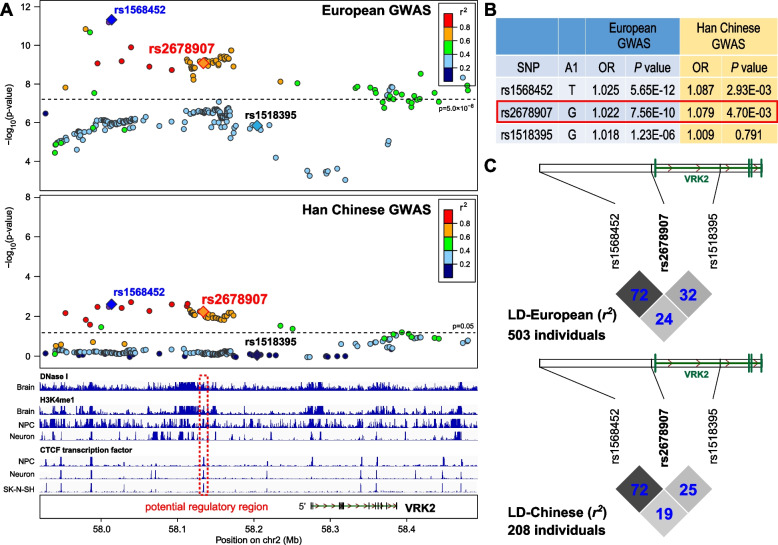
Genetic analyses of *VRK2 *with major depression. **A** Genetic associations of SNPs spanning *VRK2 *region with major depression in European GWAS, and the epigenome signatures in human brains and cell types, lead to identification of a “potential regulatory region” and a regulatory SNP (rs2678907). A physical map of the region is given and depicts known genes within the region, and the LD is defined based on the SNP rs1568452. NPC, neural progenitor cells. **B** Association results of the 3 tested *VRK2 *SNPs with major depression in European and Han Chinese samples. **C** The linkage disequilibrium (LD) maps of the 3 tested *VRK2* SNPs in European and Han Chinese individuals from 1000 Genomes Project. The figure was generated using the Haploview software, and the LD of the tested SNPs was calculated using *r*^2^ algorithm implemented in the Haploview program

We retrieved the summary statistics of *VRK2* SNPs in 23andMe, UK Biobank, and PGC2 samples [2, 3], and 256 SNPs region were available in all three GWAS datasets (a total of 246,363 cases and 561,190 controls). An overview about the strategy and logic of choosing SNPs within the *VRK2* region is provided in Figure S1 (Additional file 2). Meta-analysis found that 71 SNPs within Chr2:57,917,222–58,482,646 (hg19) region showed genome-wide significant associations with risk of depression (*P* < 5.00⨯10^–8^, Fig. 1). Among these SNPs, the lead SNP was rs1568452 in the upstream of *VRK2* (*P* = 8.12⨯10^–12^; Fig. 1A; Additional file 1: Table S1); however, the previously reported SNP rs1518395 did not achieve genome-wide significance in this meta-analysis (*P* = 1.23⨯10^–6^).

To test whether these 71 significant SNPs within Chr2:57,917,222–58,482,646 (hg19) region also showed evidence of associations with depression in Han Chinese population, we then retrieved them from a Han Chinese depression GWAS (5303 cases and 5337 controls) [8], and 47 SNPs (including rs1568452) also showed nominal associations (*P* < 0.05; Fig. 1A; Additional file 1: Table S1) and were remained for the following population genetic analyses and functional predictions. We constructed the LD map of these 47 SNPs in Europeans and Han Chinese populations using genotype data from 1000 Genomes Project [19]. The LD structure was similar between Europeans and Han Chinese (Additional file 2: Figure S2). These 47 SNPs constituted up to 3 LD blocks in Europeans and Han Chinese, and most of the SNPs in each block were in strong LD.

### The depression risk G-allele of rs2678907 decreased the enhancer activities

The above 47 SNPs were located in the noncoding regions spanning *VRK2*, and we therefore carried out the functional predictions using epigenome data (GSE96018, GSE123202, GSE100991) from ENCODE project [20] by comparing their locations with regions of open chromatin, with regions of active histone H3 lysine modifications, and with regions of ChIP-Seq data for CTCF transcription factor, we found that the genomic region spanning rs2678907 exhibited large spatial overlap with enhancer regulatory marks (e.g., H3K4me1) in multiple human cell lines (SK-N-SH, neuron, neural progenitor cells) and brain tissues (Fig. 1A), and the DNA region was demonstrated to bind CTCF and other transcription factors.

We verified the results of functional predictions using HaploReg v4.2 [21], which also utilized data primarily from ENCODE projects. In this dataset, rs2678907 was predicted to be located in the regulatory enhancer region in brain tissues (assessed by DNase I, H3K4me1 and H3K27ac marks), and ChIP-Seq experiments demonstrated that DNA regions spanning rs2678907 bound 8 proteins, including CTCF, BCLAF1, RAD21, SMC3, YY1, ZNF143, SRF, and TAL1 (Additional file 2: Figure S3). Further functional prediction using GWAVA [22] also indicated that rs2678907 was likely a functional SNP, as the prediction scores was higher than or near 0.5 (prediction scores from three different versions of the classifier (Region score, TSS score, Unmatched score) range 0–1, and higher scores suggest a greater likelihood of functionality) (Additional file 2: Figure S4).

Rs2678907 was located 1.3 kb 5’ upstream of *VRK2*, and showed genome-wide significant association with risk of depression in our meta-analysis of European samples (*P* = 7.40⨯10^–10^), and also exhibited nominal associations with in the Han Chinese GWAS (*P* = 4.70⨯10^–3^) (Fig. 1B; Additional file 1: Table S1), further strengthening its link with the illness. According to the genotype data in 1000 Genomes Project, rs2678907 was in moderate to strong LD with rs1568452 (*r*^2^ > 0.7), but both SNPs showed weak LD with rs1518395 (*r*^2^ < 0.4) (Fig. 1C).

**Fig. 2:**
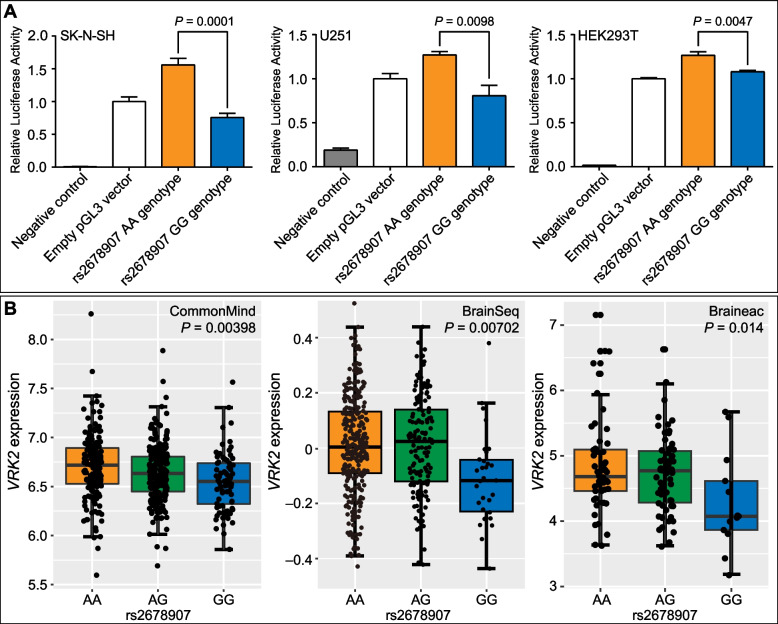
Effects of rs2678907 on enhancer activities and *VRK2* mRNA expression. **A** Results of the reporter gene assay testing the regulatory activities of rs2678907 in SK-N-SH, U251, and HEK293T cell lines. “Negative control” means pGL3 basic empty vector (which does not have promoter activity). “Empty pGL3 vector” means pGL3 promoter empty vector (which has an internal promoter). The Y-axis values represent fold changes of luciferase activity relative to the “Empty pGL3 vector” group. The means and SD of at least three biological replicates are shown. **B** Expression quantitative trait loci (eQTL) analyses of rs2678907 with *VRK2* mRNA in the CommonMind DLPFC (left), BrainSeq caudate nucleus (middle), and Braineac frontal cortex (right) tissues. The affective or bipolar cases in the eQTL datasets were not overlapped with the samples included in the meta-analysis of European GWAS samples

We then tested if the DNA fragment containing rs2678907 showed enhancer activity. The DNA fragments in the 5’ upstream of *VRK2* gene containing either the G allele or the A allele at rs2678907 were constructed into pGL3-promoter plasmid and the relative enhancer activities were measured by dual-luciferase reporter assay in human SK-N-SH, U251, and HEK293T cell lines. The enhancer activity containing the A allele was significantly higher than that of the G allele in SK-N-SH (*P* = 0.0001), U251 (*P* = 0.0098), and HEK293T (*P* = 0.0047) cell lines (Fig. 2A). These data indicated that the depression risk G-allele at rs2678907 decreased the DNA enhancer activity.

### The depression risk G-allele of rs2678907 and diagnostic status predicted lower VRK2 mRNA expression

To understand whether rs2678907 was associated with mRNA expression of nearby genes, we examined the eQTL data in human brain tissues from several independent datasets, and notably, the depression risk G-allele at rs2678907 consistently predicted lower mRNA level of *VRK2* in three independent samples (*P* = 3.98⨯10^–3^ in 467 individuals of CommonMind dataset [23]; *P* = 7.02⨯10^–3^ in 444 individuals of Caudate eQTL dataset [24]; *P* = 0.014 in 134 individuals of Braineac dataset [25]; Fig. 2B). The rs2678907 G-allele also predicted reduced mRNA expression of *FANCL* in one dataset (*P* = 2.89⨯10^–4^ in 467 individuals of CommonMind dataset [23]; Additional file 2: Figure S5). Therefore, reduced mRNA levels of *VRK2* and *FANCL* were likely associated with an increased genetic risk of depression.

We then investigated the mRNA expression of *VRK2* and *FANCL* in patients with depression and healthy controls. Intriguingly, we found that expression of *VRK2* was significantly reduced in the blood of either 882 current depression patients or 635 remitted depression patients compared with 331 healthy controls (*P* = 0.00888 for current depression versus control; *P* = 0.023 for remitted depression versus control; *P* = 0.0074 for current + remitted depression versus control) [26]. The diagnostic analysis of *VRK2* mRNA expression is consistent with the results of eQTL analysis and further confirms the involvement of *VRK2* in depression. However, *FANCL* did not alter significantly between depression patients and healthy controls (*P* = 0.199 for current depression versus control; *P* = 0.22 for remitted depression versus control; *P* = 0.17 for current + remitted depression versus control) [26] and was thus dropped out from further analyses.

### *Vrk2*-deficient mice exhibited depressive-like behaviors

**Fig. 3:**
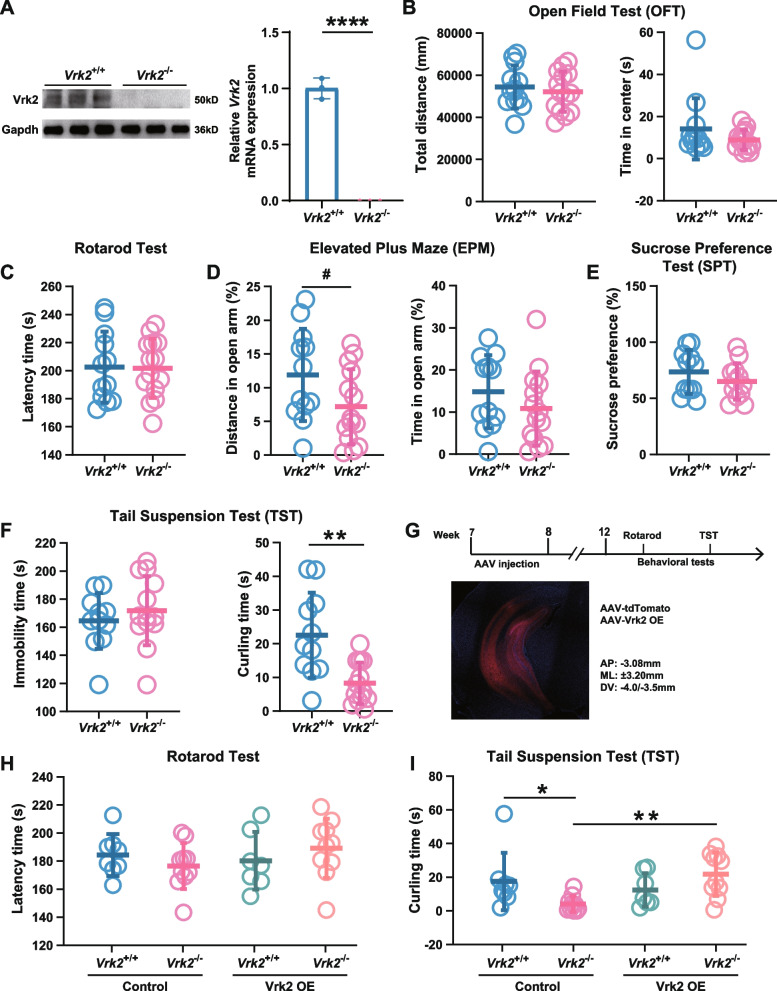
Knockout of *Vrk2* leads to depressive-like behaviors in mice. **A** knockout efficiency of *Vrk2* in the hippocampus. *n* = 3 mice/group. *P* = 0.000047. **B** Open field test. No significant difference was seen in the total distance (*P* = 0.576) and the central exploration time (*P* = 0.217) between *Vrk2*^+/+^ and *Vrk2*^−/−^ mice. *Vrk2*^+/+^, *n* = 12. *Vrk2*^−/−^, *n* = 14. **C** Rotarod test. There was no difference in latency time (*P* = 0.932) between *Vrk2*^+/+^ and *Vrk2*^−/−^ mice. *Vrk2*^+/+^, *n* = 12. *Vrk2*^−/−^, *n* = 14. **D** Elevated plus maze. The proportion of distance (*P* = 0.0640) covered by *Vrk2*^−/−^ mice in the open arm was trending downward compared with *Vrk2*^+/+^ mice, but there was no difference in the amount of time spent there (*P* = 0.258). *Vrk2*^+/+^, *n* = 12. *Vrk2*^−/−^, *n* = 14. **E** Sucrose preference test. *Vrk2*^+/+^, *n* = 11. *Vrk2*^−/−^, *n* = 12. *P* = 0.265. **F** Tail suspension test. The immobility time (*P* = 0.438) was not different, but the curling time (*P* = 0.0016) was significantly decreased in *Vrk2*^−/−^ mice. *Vrk2*^+/+^, *n* = 11. *Vrk2*^−/−^, *n* = 14. **G** Experimental timeline and schematic diagram of ventral hippocampal virus injection. **H** Rotarod test. *Vrk2*^+/+^ + ctrl, *n* = 8; *Vrk2*^−/−^ + ctrl, *n* = 10; *Vrk2*^+/+^ + Vrk2, *n* = 7; *Vrk2*^−/−^ + Vrk2, *n* = 10. *Vrk2*^+/+^ + ctrl vs. *Vrk2*^−/−^ + ctrl, *P* = 0.380; *Vrk2*^+/+^ + ctrl vs. *Vrk2*^−/−^ + Vrk2, *P* = 0.582; *Vrk2*^−/−^ + ctrl vs. *Vrk2*^−/−^ + Vrk2, *P* = 0.135. **I** Tail suspension test. *Vrk2*^−/−^ + Vrk2 mice showed a considerably greater curling time compared with *Vrk2*^−/−^ + ctrl mice (*Vrk2*^+/+^ + ctrl vs. *Vrk2*^−/−^ + ctrl, *P* = 0.0229; *Vrk2*^+/+^ + ctrl vs. *Vrk2*^−/−^ + Vrk2, *P* = 0.452; *Vrk2*^−/−^ + ctrl vs. *Vrk2*^−/−^ + Vrk2, *P* = 0.0022). *Vrk2*^+/+^ + ctrl, *n* = 8; *Vrk2*^−/−^ + ctrl, *n* = 10; *Vrk2*^+/+^ + Vrk2, *n* = 7; *Vrk2*^−/−^ + Vrk2, *n* = 10. Two-tailed *t*-test for **A**–**F**. One-way ANOVA for **H** and **I**. ^#^*P* < 0.1, *P < 0.05, **P < 0.01, ***P < 0.001. Error bars indicate mean ± SD

We conducted behavioral analyses using *Vrk2*^−/−^ and wild-type (*Vrk2*^+/+^) mice. Both types of mice were male littermates 8–10 weeks old, and *Vrk2* expression was nonvisible in the *Vrk2*^−/−^ mice (Fig. 3A; Additional file 2: Figure S6). In our results, *Vrk2*^−/−^ mice did not show significantly higher exploratory activity in OFT than *Vrk2*^+/+^ mice (*Vrk2*^+/+^, 54,388 ± 10,253 mm distance; *Vrk2*^−/−^, 52,148 ± 9558 mm distance, *df* = 24, *t* = 0.576, *P* = 0.570, *n* = 12–14; Fig. 3B), and there were no differences between experimental groups for the time spent in the center of arena (*Vrk2*^+/+^, 14.09 ± 14.57 s in center arena; *Vrk2*^−/−^, 8.87 ± 4.71 s in center arena, *df* = 24, *t* = 1.269, *P* = 0.217, *n* = 12–14; Fig. 3B). Rotarod test was used to determine whether deficiency of *Vrk2* affects motor ability; however, no significant difference was observed (*Vrk2*^+/+^, 202.50 ± 25.36 s in latency time; *Vrk2*^−/−^, 201.70 ± 21.07 s in latency time, *df* = 24, *t* = 0.0867, *P* = 0.932, *n* = 12–14; Fig. 3C). In EPM, no significant differences were found in the percentage of time (*Vrk2*^+/+^, 14.83 ± 8.70% time in open arms; *Vrk2*^−/−^, 10.84 ± 8.81% time in open arms, *df* = 24, *t* = 1.159, *P* = 0.258, *n* = 12–14; Fig. 3D) and distance (*Vrk2*^+/+^, 11.89 ± 6.85% distance in open arms; *Vrk2*^−/−^, 7.16 ± 5.57% distance in open arms, *df* = 24, *t* = 1.942, *P* = 0.0640, *n* = 12–14; Fig. 3D) that the mice entered the open arms. There was no difference in sucrose preference between *Vrk2*^−/−^ and *Vrk2*^+/+^ mice (*Vrk2*^+/+^, 73.44 ± 19.51% sucrose preference; *Vrk2*^−/−^, 64.91 ± 16.16% sucrose preference, *df* = 21, *t* = 1.146, *P* = 0.2648, *n* = 11–12; Fig. 3E). In TST, although no difference in the immobility time was observed between genotypic groups (*Vrk2*^+/+^, 164.50 ± 19.89 s in immobility time; *Vrk2*^−/−^, 171.80 ± 24.58 s in immobility time, *df* = 22, *t* = 0.790, *P* = 0.438, *n* = 11–13; Fig. 3F), the *Vrk2*^−/−^ mice exhibited significantly reduced time in curling (*Vrk2*^+/+^, 22.53 ± 12.63 s in curling time; *Vrk2*^−/−^, 8.28 ± 6.15 s in curling time, *df* = 22, *t* = 3.606, *P* = 0.0016, *n* = 11–13; Fig. 3F), a more active escape-oriented behavior. These results suggest that *Vrk2*^−/−^ mice might exhibit depressive-like behaviors compared with *Vrk2*^+/+^ mice.

Subsequently, we investigated whether overexpressing Vrk2 could rescue the depressive-like behaviors in *Vrk2*^−/−^ mice. We respectively injected Vrk2 overexpressing AAV and control virus into the ventral hippocampus of *Vrk2*^−/−^ and *Vrk2*^+/+^ mice at 8-weeks old and performed rotarod test and TST four weeks later (Fig. 3G). The chosen ventral hippocampus for microinjection is based on previous studies, in which they demonstrated that the ventral hippocampus is more connected to stress, mood, and emotion than the dorsal hippocampus [39, 40] and seems to be more closely linked to the processing of emotional information [41, 42]. We observed no change in motor ability between the experimental groups(*Vrk2*^+/+^ + ctrl, 184.30 ± 14.96 s in latency time, *n* = 8; *Vrk2*^−/−^ + ctrl, 176.60 ± 16.43 s in latency time, *n* = 10; *Vrk2*^+/+^ + Vrk2, 180.20 ± 20.62 s in latency time,* n* = 7; *Vrk2*^−/−^ + Vrk2, 189.20 ± 21.04 s in latency time,* n* = 10; *Vrk2*^+/+^ + ctrl vs. *Vrk2*^−/−^ + ctrl, *df* = 31, *t* = 0.891, *P* = 0.380; *Vrk2*^−/−^ + ctrl vs. *Vrk2*^−/−^ + Vrk2, *df* = 31, *t* = 1.535, *P* = 0.135; multiple comparisons using Fisher’s LSD in one-way ANOVA; Fig. 3H); however, we found that the curling time of *Vrk2*^−/−^ mice after overexpressing Vrk2 in the ventral hippocampus was significantly enhanced **(***Vrk2*^+/+^ + ctrl, 17,51 ± 17.04 s in curling time, *n* = 8; *Vrk2*^−/−^ + ctrl, 4.07 ± 4.72 s in curling time, *n* = 10; *Vrk2*^+/+^ + Vrk2, 12.38 ± 9.99 s in curling time,* n* = 7; *Vrk2*^−/−^ + Vrk2, 21.78 ± 12.95 s in curling time,* n* = 10; *Vrk2*^+/+^ + ctrl vs. *Vrk2*^−/−^ + ctrl, *df* = 31, *t* = 2.394, *P* = 0.0229; *Vrk2*^−/−^ + ctrl vs. *Vrk2*^−/−^ + Vrk2, *df* = 31, *t* = 3.346, *P* = 0.0022; multiple comparisons using Fisher’s LSD in one-way ANOVA; Fig. 3I). These results imply that overexpression of Vrk2 in the ventral hippocampus of *Vrk2*^−/−^ mice may have antidepressant effect.

### Decreased *Vrk2* expression in mice ventral hippocampus resulted in depressive-like behaviors

**Fig. 4:**
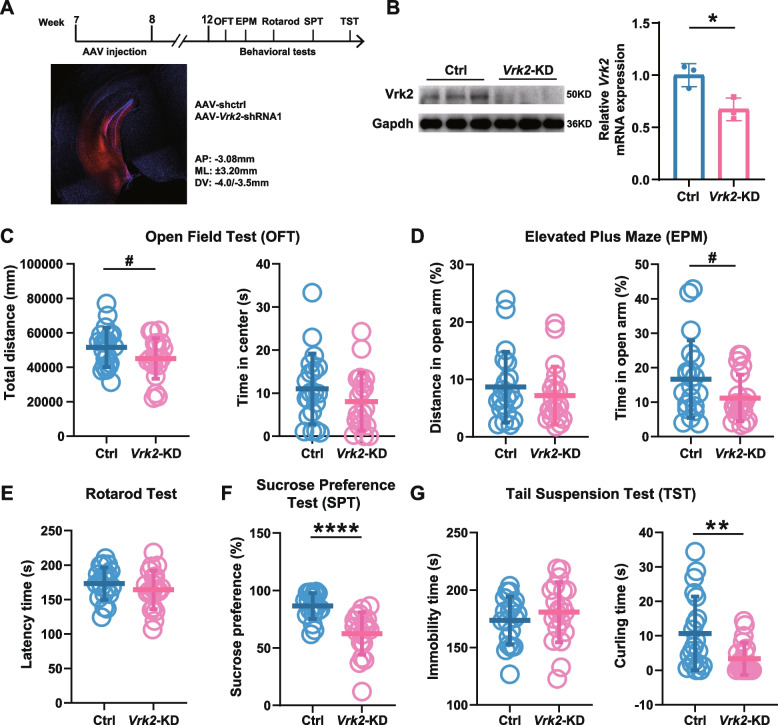
Decreased *Vrk2 *expression in the hippocampus results in depressive-like behaviors in mice. **A** Experimental timeline and schematic diagram of ventral hippocampal virus injection. **B** The shRNA mediated *Vrk2* knockdown (KD) efficiency in the ventral hippocampus. *n* = 3 mice/group. *P* = 0.0211. **C** Open field test. No effects were found in the total distance (*P* = 0.0907) and time spent in center (*P* = 0.219). Ctrl, *n* = 20. *Vrk2*-KD, *n* = 20. **D** Elevated plus maze. Decreasing trend in the percentage of time (*P* = 0.0698) that *Vrk2*-KD mice entering the open arms while the distance ratio (*P* = 0.405) into the open arms remains constant. Ctrl, *n* = 20. *Vrk2*-KD, *n* = 20. **E** Rotarod test. Ctrl, *n* = 20. *Vrk2*-KD, *n* = 20. *P* = 0.284. **F** Sucrose preference test. The percentage of sucrose preference (*P* < 0.0001) significantly decreased in *Vrk2*-KD mice. Ctrl, *n* = 20. *Vrk2*-KD, *n* = 20. **G** Tail suspension test. The immobility time (*P* = 0.351) was no difference, but the curling time (*P* = 0.0079) was significantly decreased in *Vrk2*-KD mice. Ctrl, *n* = 20. *Vrk2*-KD, *n* = 20. ^#^*P* < 0.1,* P < 0.05, ** P < 0.01, *** P < 0.001 (two-tailed *t*-test). Error bars indicate mean ± SD

To further validate the behavioral phenotypes observed in the *Vrk2*^−/−^ mice, we specifically knocked down *Vrk2* in the ventral hippocampus of wild-type C57BL/6 J mice using adeno-associated virus (AAV) packaged shRNA (Fig. 4A). We designed three shRNA sequences respectively targeting the *Vrk2* gene and examined their knockdown efficiencies, and the shRNA1 showing the strongest suppressive effects was selected (Additional file 2: Figure S7). Four weeks after bilateral microinjection of AAV into the ventral hippocampus, the *Vrk2* knockdown efficiency was verified (Fig. 4B) and then behavioral tests were carried out. In OFT, no significant difference was observed in the total distance (control, 51,464 ± 11,418 mm distance; *Vrk2*-KD, 45,080 ± 11,837 mm distance, *df* = 38, *t* = 1.736, *P* = 0.0907, *n* = 20–20; Fig. 4C) and central exploration time (control, 11.00 ± 8.22 s in center arena; *Vrk2*-KD, 8.03 ± 6.76 s in center arena, *df* = 38, *t* = 1.249, *P* = 0.219, *n* = 20–20; Fig. 4C) between shRNA-mediated *Vrk2* knockdown (*Vrk2*-KD) and the mice injected with control shRNA AAV (control). The proportion of time (control, 16.67 ± 11.26% time in open arms; *Vrk2*-KD, 11.19 ± 6.72% time in open arms, *df* = 38, *t* = 1.866, *P* = 0.0698, *n* = 20–20; Fig. 4D) and distance (control, 8.67 ± 6.16% distance in open arms; *Vrk2*-KD, 7.17 ± 5.05% distance in open arms, *df* = 38, *t* = 0.843, *P* = 0.405, *n* = 20–20; Fig. 4D) that the mice entered the open arms had no influence in EPM, and there was also no difference in motor ability between the groups in rotarod test (control, 173.00 ± 23.49 s in latency time; *Vrk2*-KD, 164.10 ± 28.02 s in latency time, *df* = 38, *t* = 1.087, *P* = 0.284, *n* = 20–20; Fig. 4E). In SPT, *Vrk2*-KD mice showed significantly lower sucrose preference than control mice (control, 86.60 ± 11.19% sucrose preference; *Vrk2*-KD, 62.54 ± 18.39% sucrose preference, *df* = 38, *t* = 4.998, *P* < 0.0001, *n* = 20–20; Fig. 4F). In TST, the *Vrk2*-KD mice spent less time in curling behavior compared with control mice (control, 10.67 ± 10.71 s in curling time; Vrk2-KD, 3.34 ± 4.70 s in curling time, *df* = 38, *t* = 2.804, *P* = 0.0079, *n* = 20–20; Fig. 4G). These results indicate that decreased *Vrk2* in the ventral hippocampus leads to depressive-like behaviors in mice and also in line with the previous study showing that activation of the ventral hippocampal projections to the nucleus accumbens projections increases sensitivity to stress while decreasing the sucrose preference [43].

### Defective *Vrk2* results in loss of mushroom spines in the ventral hippocampus

**Fig. 5:**
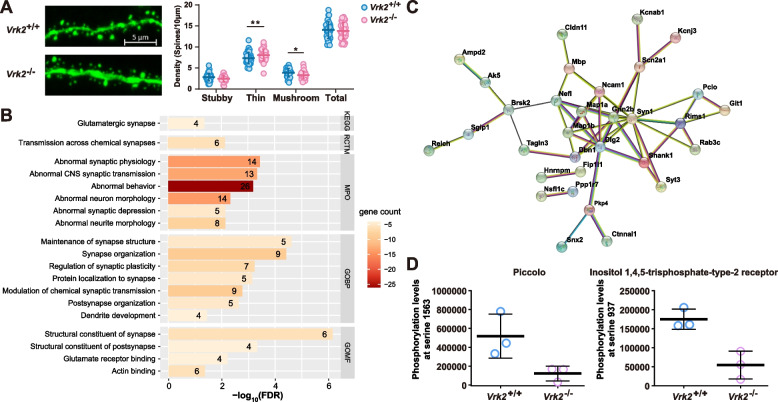
Reduced mushroom spine and modifications to synapse-associated proteins in the ventral hippocampus of *Vrk2*^−/−^ mice. **A** Dendritic spine density and morphological analysis of *Vrk2*^−/−^ mice ventral hippocampus pyramidal neurons. Scale bars represent 5 μm. With no difference in total density (*P* = 0.664), *Vrk2*^−/−^ mice showed a much lower number of mushroom spines (*P* = 0.0265) and a significantly higher number of thin spines (*P* = 0.0039). *Vrk2*^+/+^, *n* = 37; *Vrk2*^−/−^, *n* = 40. Two-tailed *t*-tests were used to compare the total number of dendritic spines, and two-way ANOVA was used to determine the various morphologies. * P < 0.05, ** P < 0.01. **B** KEGG pathway and GO analyses of the 50 significantly down-regulated phosphorylated proteins in *Vrk2*^−/−^ mice. **C** Protein–protein interaction (PPI) networks of the 50 significantly down-regulated phosphorylated proteins in *Vrk2*^−/−^ mice. **D** Pclo phosphorylation at serine 1563 and Itpr2 phosphorylation at serine 937 were significantly downregulated after deletion of *Vrk2* (*P* = 0.049 for Pclo and *P* = 0.010 for Itpr2, two-tailed *t*-test). *n* = 3 per group. Error bars indicate mean ± SD

The results of neurobiological and histopathological analyses have demonstrated that depression is associated with synaptic dysfunction and loss of dendritic spines in brain regions associated with emotional and cognitive regulation, such as the hippocampus [44, 45]. Dendritic spines are the postsynaptic sites of most excitatory synapses, which are the basic units of connection and information exchange between neurons and are intimately associated with emotional and cognitive functions [46, 47]. Notably, alteration in the morphogenesis of dendritic spines is one of the key pathological characteristics of depression [48]. Therefore, we evaluated the effect of *Vrk2* deficiency on the density and morphology of dendritic spines in mice. The neuronal sparse labeling results of pyramidal neurons in the ventral hippocampus showed no difference in the density of total dendritic spines between *Vrk2*^+/+^ and *Vrk2*^−/−^ mice (*Vrk2*^+/+^, 14.030 ± 2.023 spines per 10 μm; *Vrk2*^−/−^, 13.840 ± 1.944 spines per 10 μm, *df* = 75, *t* = 0.436, *P* = 0.664, two-tailed *t*-test; Fig. 5A). The morphological analysis revealed that compared to wild-type mice, deletion of *Vrk2* induced a significant decrease in the density of mushroom spines (*Vrk2*^+/+^, 3.868 ± 1.007 spines per 10 μm; *Vrk2*^−/−^, 3.313 ± 0.924 spines per 10 μm, *df* = 225, *t* = 2.233, *P* = 0.0265, multiple comparisons using Fisher’s LSD in two-way ANOVA; Fig. 5A), the most mature type of spines that play vital roles in synaptic transmission and plasticity, and a significant increase in the density of thin spines (*Vrk2*^+/+^, 7.348 ± 1.380 spines per 10 μm; *Vrk2*^−/−^, 8.073 ± 1.420 spines per 10 μm, *df* = 225, *t* = 2.916, *P* = 0.0039, multiple comparisons using Fisher’s LSD in two-way ANOVA; Fig. 5A). Our results demonstrated that *Vrk2* knockout can cause a loss of mushroom spines and an increase in thin spines in the ventral hippocampus.

### Deletion of *Vrk2* affects synapse-associated proteins and processes in the ventral hippocampus

To investigate the molecular mechanisms underlying the depressive-like behaviors in *Vrk2*^−/−^ mice, we performed phosphorylated 4D-label-free quantitative proteomics analyses of the ventral hippocampal proteins in *Vrk2*^−/−^ and *Vrk2*^+/+^ mice (*n* = 3 each). After stringent quality control, 6658 quantifiable phosphorylated peptides were identified in 2847 phosphorylated proteins. The distribution of phosphorylation modification sites revealed that serine (S, 79.9%) and threonine (T, 14.85%) were most commonly phosphorylated. A total of 98 differentially phosphorylated peptides with an absolute fold change (FC) > 2 (|log2(FC)|> 1) at *P* < 0.05 between *Vrk2*^−/−^ and *Vrk2*^+/+^ mice were identified (Additional file 1: Table S2). Among them, 56 (mapped to 50 proteins) and 42 peptides (mapped to 38 proteins) had significantly lower and higher phosphorylation levels, respectively, following the deletion of *Vrk2*.

For 38 proteins with highly phosphorylated peptides, KEGG pathway and GO analyses did not reveal any significant enrichment pathways or biological processes. However, for the 50 proteins with lower phosphorylated peptides, KEGG pathway analysis revealed significantly enriched signals in glutamatergic synapse. Reactome pathway analysis demonstrated that these were significantly enriched in the pathways of “transmission across chemical synapses”. Furthermore, Monarch HPO analysis revealed that those proteins were enriched in terms including “abnormal synaptic physiology,” “abnormal behavior,” and “abnormal neuron morphology” (Fig. 5B). Additionally, gene set enrichment analyses (GSEA) indicated that these proteins were strongly enriched in the GO terms “maintenance of synapse structure,” “postsynapse organization,” “regulation of synaptic plasticity,” and “structural constituent of synapse” etc. (Fig. 5B). These 50 proteins also formed dense protein–protein interaction (PPI) networks (enrichment *P* < 1.00⨯10^−16^, Fig. 5C), and notably, 11 proteins were located at postsynaptic density, including Pkp4, Map 1a, Map 1b, Dlg2, Dbn1, Psd3, Shank1, Grin2b, Syn1, Rims1, and Pclo. In conclusion, our findings show that the deletion of *Vrk2* primarily affects synapse-associated proteins and processes.

In addition to highlighting essential pathways, phosphoproteomic analyses revealed several proteins, such as Pclo and Itpr2, whose involvement in depression has been supported by strong evidence. *Pclo* (encoding piccolo) has been implicated as a depression susceptibility gene in the early GWAS and follow-up replication studies [49–51], and mice overexpressing the C2A domain of piccolo exhibited depressive-like behaviors in forced swim and tail suspension tests [52]. *Itpr2* encodes the inositol 1,4,5-trisphosphate-type-2 receptor. Mice lacking Itpr2 exhibit deficiencies in astrocytic ATP release, abnormal rsfMRI functional connectivity (rsFC) in depression-related networks, and depressive-like behaviors, and which can be rescued by the administration of ATP [53, 54]. Pclo and Itpr2 phosphorylation at serine 1563 and 937, respectively, were significantly downregulated after the deletion of *Vrk2* (*P* = 0.049 for Pclo and *P* = 0.010 for Itpr2, Fig. 5D). This study provides additional evidence suggesting pivotal roles of Pclo and Itpr2 in depression pathogenesis.

## Discussion

Our study identified a functional SNP, rs2678907, within *VRK2* that showed consistent effects on the risk of depression across distinct populations. The risk allele of rs2678907 reduced the enhancer activity and gene expression levels of *VRK2*, which is consistent with the observed downregulation of *VRK2* mRNA expression in patients with depression. We further demonstrated that *Vrk2*^−/−^ mice exhibited a reduced curling time in the TST compared to *Vrk2*^+/+^ mice. Since curling behavior is considered a positive escape-avoidance behavior, the decrease in curling time suggests that the loss of *Vrk2* might contribute to depressive-like behavior [32]. We also subjected *Vrk2*^*−/−*^ and *Vrk2*^+/+^ mice to chronic restraint stress, and *Vrk2*^*−/−*^ mice showed reduced curling time in the TST compared to *Vrk2*^+/+^ mice (Additional file 2: Figure S8).

Antidepressant effects were observed following overexpression of *Vrk2* in the ventral hippocampus of *Vrk2*^−/−^ mice. Additionally, we also suppressed *Vrk2* in the ventral hippocampus of wild-type C57BL/6 J male mice and found that *Vrk2*-KD mice exhibited depressive-like behaviors in the SPT and TST. This specific decrease of *Vrk2* in the ventral hippocampus even caused more significant depressive-like behaviors compared with those observed in the *Vrk2*^−/−^ mice, suggesting that deficiency of *Vrk2* in the ventral hippocampus is essential in the pathogenesis of depression. We hypothesize that the reason for this difference may be because local knockdown of *Vrk2* in the ventral hippocampus affected some particular neural circuits or neural projections (to the nucleus accumbens [43], for example). However, *Vrk2*^±^ mice did not exhibit depressive-like behaviors in the SPT and TST compared to *Vrk2*^+/+^ mice (Additional file 2: Figure S9). This was not completely unexpected, and we speculated that there may be some compensatory mechanisms in *Vrk2*^±^ mice, as this gene is widely expressed throughout the brain and may have different functions in various brain regions.

Abnormalities in the density and morphology of pyramidal neuron dendritic spines in the hippocampal brain areas are frequently associated with depressive-like behavior [55]. Furthermore, we examined the dendritic spines of pyramidal neurons in the ventral hippocampus and discovered that *Vrk2*^−/−^ mice had a significant decrease in mushroom spines and a concomitant increase in thin spines, which are considered immature and usually forms unstable and weakly connected synapses [56, 57]. Lee et al. discovered that deleting *Vrk2* increased the number of pre-synapses in the hippocampus of P15 mice, but had no effect on the number of post-synapses, whereas there were no abnormalities in the number of synapses in 3-month-old mice [13]. This observation is in line with our results showing that *Vrk2* deletion had no effect on the overall dendritic spines in the ventral hippocampus. Additionally, we also performed phosphorylated proteomic analyses and found that the dysregulated proteins were mainly enriched in synapse-related functions and pathways, which warrants further studies to fully understand their mechanisms of action.

## Limitations

The present study has several limitations. First, in our SPT behavioral analysis, *Vrk2*-KD mice consumed much less sucrose solution than control mice, whereas *Vrk2*^*−/−*^ mice showed no significant difference compared to *Vrk2*^+/+^ mice. Such inconsistencies drove us to speculate that the loss of *Vrk2* in brain regions other than the ventral hippocampus caused compensatory effects since *Vrk2* also exerts functions in other brain regions [11], but this awaits further investigations; in addition, despite we focused on the hippocampus in the present study, we cannot exclude the possibility that other brain regions also participated in the pathogenesis of depression mediated by *Vrk2*, which deserves further studies. Second, *VRK2* SNPs also exhibit significant genome-wide associations with schizophrenia and bipolar disorder [58–60], and the mRNA of *VRK2* was significantly reduced in patients with schizophrenia and patients with bipolar disorder compared to controls [61]. Therefore, *VRK2* may be a universal risk gene for multiple psychiatric disorders [10], and the results of the present study may also be able to provide insights into the neurobiological mechanisms of other psychiatric disorders.

## Conclusions

Taken together, we provide direct evidence that *Vrk2* could affect depressive-like behaviors in mice, which is in line with the observation of reduced *VRK2* in depression patients compared with controls. Our results suggest that *VRK2* is a risk gene for depression, providing potential clues for its involvement in the pathogenesis of depression.

## Supplementary Information

**Additional file 1.** **Table S1. **Association results of the VRK2 SNPs withdepression in Europeans (PGC2, UK Biobank and 23andMe) and Han Chinese(CONVERGE) GWAS. **TablesS2**. Overviewof the differentially phosphorylated proteins between *Vrk2*^-/-^and *Vrk2*^+/+^ mice. 

**Additional file 2.** **Figure S1.** Flow chart ofthe genetic analyses. **Figure S2. **The linkage disequilibrium (LD) maps of the*VRK2* SNPs in European and Han Chinese individuals from 1000 GenomesProject. **Figure S3. ** Functional prediction of the 47 SNPs usingHaploReg v.4.2. **Figure S4**. Functional prediction of the 47 SNPs usingGWAVA. **Figure S5. **Expression quantitative trait loci (eQTL)analyses of rs2678907 with *FANCL* mRNAin the DLPFC tissues from CommonMind Consortium dataset. **Figure S6. **A. Knockdown efficiency of Vrk2 in thewild-type C57BL/6J male mice injected with *Vrk2*-shRNA AAV in the ventralhippocampus. B. Knockout efficiency of Vrk2 in *Vrk2*^-/-^ mice. C. Overexpression efficiency of Vrk2 in the ventralhippocampus of *Vrk2*^-/-^ mice injected with Vrk2 overexpressingAAV. **Figure S7.** Knockdown efficiency of Vrk2 mediated byshRNAs. **Figure S8. **Behavioral analyses of *Vrk2*^+/+^and *Vrk2*^-/-^ mice after chronic restraint stress (CRS). **FigureS9.** Behavioral analyses of *Vrk2*^+/+^, *Vrk2*^+/-^and *Vrk2*^-/-^ mice.

## Data Availability

All the GWAS data and statistical software used in this study were publicly available (which can be accessed through the following URLs), and all the generated results in this study were provided in the main text and supplemental data. URLs: ENCODE: https://www.encodeproject.org/data-standards/ MaxQuant 1.6.14 software: https://www.maxquant.org/ GWAS summary statistics of UK Biobank and PGC2: https://datashare.is.ed.ac.uk/handle/10283/3203 LocusZoom: http://locuszoom.sph.umich.edu/locuszoom/ Summary statistics of *VRK2* SNPs in 23andMe sample: https://www.nature.com/articles/ng.3623#Sec23 PLINK v1.9: https://zzz.bwh.harvard.edu/plink/ HaploReg v4.2: https://pubs.broadinstitute.org/mammals/haploreg/haploreg.php GWAVA: http://www.sanger.ac.uk/sanger/StatGen_Gwava CommonMind dataset: https://www.synapse.org/#!Synapse:syn2759792 Caudate eQTL dataset: http://erwinpaquolalab.libd.org/caudate_eqtl/ Braineac dataset: http://www.braineac.org/

## Abbreviations

AAV: Adeno-associated virus
ANOVA: Analysis of variance
cDNA: Complementary DNA
EPM: Elevated plus maze
eQTL: Expression quantitative trait loci
GO: Gene Ontology
GWAS: Genome-wide association studies
GWAVA: Genome-wide annotation of variants
HPO: Human phenotype ontology
KEGG: Kyoto encyclopedia of genes and genomes
KO: Knockout
LD: Linkage disequilibrium
OR: Odds ratio
PPI: Protein–protein interaction
PVDF: Polyvinylidene difluoride
RIN: RNA integrity number
RT-qPCR: Real-time quantitative PCR
SD: Standard deviation
SE: Standard error
shRNA: Short hairpin RNA
SNP: Single nucleotide polymorphism
SPT: Sucrose preference test
SVs: Surrogate variables
TST: Tail suspension test
UKBEC: UK brain expression consortium
VRK2: VRK serine/threonine kinase 2
