# Supplementary material for: Reduced Vrk2 expression is associated with higher risk of depression in humans and mediates depressive-like behaviors in mice
Source: BMC Med. 2023 Jul 14;21:256. doi: 10.1186/s12916-023-02945-0 (PMC10349461; doi:10.1186/s12916-023-02945-0)
Supplement: Supplementary file 1 — Additional file 1. Table S1. Association results of the VRK2 SNPs withdepression in Europeans (PGC2, UK Biobank and 23andMe) and Han Chinese(CONVERGE) GWAS. TablesS2. Overviewof the differentially phosphorylated proteins between Vrk2-/-and Vrk2+/+ mice. [file 12916_2023_2945_MOESM1_ESM.pdf]

Table S1. Association results of the *VRK2* SNPs with depression in Europeans (PGC2 [1], UK Biobank [2] and 23andMe [3]) and Han Chinese (CONVERGE [4]) GWAS.

CHR	POS	SNP	A1	A2	Frequency of A1		PGC2+UKB			23andMe			PGC2+UKB+23andMe meta-analysis				CONVERGE		
					European	Chinese	OR	SE	*P*	OR	SE	*P*	*P*	OR	Q	I	OR	SE	*P*
2	57956088	rs13025126	A	G	0.385	0.411	1.017	0.0044	1.16E-04	1.027	0.0062	1.55E-05	1.34E-08	1.021	0.197	39.82	1.081	0.028	6.02E-03
2	57987593	rs11682175	T	C	0.528	0.558	0.981	0.0043	6.91E-06	0.968	0.0060	9.10E-08	1.10E-11	0.977	0.083	66.74	0.935	0.028	1.57E-02
2	57988194	rs11688767	A	T	0.514	0.540	0.981	0.0043	8.84E-06	0.969	0.0060	1.39E-07	1.86E-11	0.977	0.088	65.68	0.939	0.028	2.29E-02
2	57995348	rs13016665	A	C	0.429	0.580	1.016	0.0044	4.27E-04	1.029	0.0062	4.54E-06	3.32E-08	1.020	0.090	65.27	1.077	0.029	2.04E-02
2	57998040	rs11695135	A	C	0.610	0.582	0.981	0.0044	2.24E-05	0.972	0.0062	3.59E-06	7.55E-10	0.978	0.197	39.82	0.920	0.028	2.95E-03
2	58012833	rs1568452	T	C	0.381	0.413	1.022	0.0044	1.51E-06	1.032	0.0062	3.19E-07	5.65E-12	1.025	0.180	44.44	1.087	0.028	2.93E-03
2	58029645	rs2717066	A	C	0.371	0.414	1.019	0.0045	2.58E-05	1.030	0.0062	1.77E-06	5.83E-10	1.023	0.155	50.6	1.085	0.028	3.33E-03
2	58041936	rs2717046	T	C	0.378	0.409	1.020	0.0044	7.95E-06	1.030	0.0062	1.43E-06	1.11E-10	1.023	0.188	42.2	1.092	0.028	1.70E-03
2	58065936	rs2717079	A	T	0.390	0.453	1.019	0.0044	1.96E-05	1.028	0.0061	9.39E-06	1.11E-09	1.022	0.270	17.88	1.082	0.028	4.73E-03
2	58095252	rs2717067	C	G	0.393	0.430	1.019	0.0044	2.79E-05	1.028	0.0061	9.11E-06	1.66E-09	1.022	0.242	26.95	1.089	0.028	2.20E-03
2	58111064	rs6720288	A	G	0.617	0.573	0.981	0.0044	9.99E-06	0.973	0.0062	6.56E-06	4.73E-10	0.978	0.281	14.04	0.920	0.028	2.68E-03
2	58111922	rs1533725	T	G	0.617	0.577	0.981	0.0044	1.01E-05	0.973	0.0062	7.65E-06	6.00E-10	0.978	0.287	11.9	0.917	0.028	2.06E-03
2	58115072	rs2678911	C	G	0.383	0.458	1.020	0.0044	1.02E-05	1.028	0.0062	8.36E-06	6.75E-10	1.022	0.299	7.39	1.081	0.028	4.18E-03
2	58116640	rs2683632	T	C	0.615	0.540	0.981	0.0044	1.65E-05	0.973	0.0062	8.19E-06	1.14E-09	0.978	0.264	20	0.929	0.028	5.93E-03
2	58117095	rs2678913	T	C	0.615	0.542	0.981	0.0044	2.03E-05	0.973	0.0062	8.21E-06	1.44E-09	0.979	0.253	23.64	0.928	0.028	5.64E-03
2	58117926	rs2678914	C	G	0.615	0.542	0.981	0.0044	1.91E-05	0.973	0.0062	8.04E-06	1.28E-09	0.979	0.258	21.85	0.928	0.028	5.64E-03
2	58119122	rs2717060	A	G	0.617	0.542	0.982	0.0044	4.46E-05	0.973	0.0062	8.36E-06	3.37E-09	0.979	0.221	33.17	0.928	0.028	5.59E-03
2	58119582	rs2678916	T	G	0.384	0.456	1.019	0.0044	1.64E-05	1.028	0.0062	9.80E-06	1.21E-09	1.022	0.287	11.9	1.076	0.028	7.32E-03
2	58119657	rs2717059	C	G	0.618	0.544	0.982	0.0044	6.21E-05	0.973	0.0062	8.29E-06	5.28E-09	0.979	0.202	38.57	0.929	0.028	7.31E-03
2	58120553	rs2678917	A	T	0.385	0.456	1.019	0.0044	1.94E-05	1.028	0.0062	8.61E-06	1.36E-09	1.022	0.264	20	1.076	0.028	7.32E-03
2	58121391	rs2678918	T	C	0.384	0.456	1.019	0.0044	1.82E-05	1.028	0.0062	9.15E-06	1.28E-09	1.022	0.275	16.1	1.075	0.028	7.59E-03
2	58127907	rs2717057	T	C	0.382	0.456	1.019	0.0044	1.50E-05	1.029	0.0062	4.92E-06	5.98E-10	1.023	0.237	28.64	1.079	0.028	4.99E-03
2	58133522	rs2678907	A	G	0.618	0.542	0.981	0.0044	1.83E-05	0.972	0.0062	4.64E-06	7.56E-10	0.978	0.226	31.71	0.927	0.028	4.70E-03
2	58135023	rs867740	T	C	0.617	0.544	0.981	0.0044	1.36E-05	0.972	0.0062	4.88E-06	5.01E-10	0.978	0.237	28.64	0.932	0.028	9.00E-03
2	58136220	rs2717000	C	G	0.617	0.540	0.981	0.0044	1.36E-05	0.972	0.0062	5.05E-06	5.64E-10	0.978	0.247	25.36	0.933	0.028	9.91E-03
2	58138581	rs2678902	A	G	0.383	0.460	1.019	0.0044	1.92E-05	1.028	0.0062	5.90E-06	1.01E-09	1.022	0.237	28.64	1.071	0.028	1.06E-02
2	58139906	rs2678895	A	T	0.618	0.544	0.981	0.0044	1.22E-05	0.973	0.0062	6.61E-06	5.99E-10	0.978	0.269	18.08	0.930	0.028	7.12E-03
2	58144542	rs951965	A	C	0.383	0.456	1.020	0.0044	1.14E-05	1.028	0.0062	6.00E-06	5.65E-10	1.023	0.264	20	1.074	0.028	7.61E-03
2	58145060	rs2717005	A	G	0.619	0.540	0.981	0.0044	1.30E-05	0.973	0.0062	7.03E-06	7.15E-10	0.978	0.269	18.08	0.934	0.028	1.08E-02
2	58148349	rs2465805	T	C	0.382	0.460	1.019	0.0044	1.31E-05	1.028	0.0062	5.73E-06	5.99E-10	1.023	0.253	23.64	1.071	0.028	1.03E-02
2	58149158	rs2717008	T	C	0.618	0.540	0.981	0.0044	1.17E-05	0.972	0.0062	5.81E-06	5.32E-10	0.978	0.258	21.85	0.934	0.028	1.06E-02
2	58152280	rs2717012	A	C	0.382	0.460	1.019	0.0044	1.49E-05	1.028	0.0062	8.76E-06	9.58E-10	1.022	0.281	14.04	1.070	0.028	1.14E-02
2	58152537	rs2717013	A	G	0.382	0.460	1.019	0.0044	1.59E-05	1.028	0.0062	6.21E-06	8.03E-10	1.022	0.247	25.36	1.070	0.028	1.17E-02
2	58154915	rs2678874	A	T	0.378	0.462	1.020	0.0044	7.69E-06	1.028	0.0062	6.07E-06	3.31E-10	1.023	0.281	14.04	1.069	0.028	1.26E-02
2	58155829	rs2312143	A	G	0.378	0.463	1.020	0.0044	9.18E-06	1.028	0.0062	6.32E-06	3.96E-10	1.023	0.281	14.04	1.069	0.028	1.31E-02
2	58159852	rs2717020	T	C	0.622	0.540	0.980	0.0044	7.75E-06	0.972	0.0062	6.05E-06	3.31E-10	0.978	0.281	14.04	0.936	0.028	1.32E-02
2	58160138	rs2678885	T	G	0.622	0.535	0.981	0.0044	1.01E-05	0.973	0.0062	7.11E-06	4.73E-10	0.978	0.281	14.04	0.932	0.028	8.49E-03
2	58160605	rs2717021	A	G	0.378	0.456	1.020	0.0044	9.84E-06	1.028	0.0062	6.00E-06	4.20E-10	1.023	0.269	18.08	1.073	0.028	8.72E-03
2	58163146	rs2717025	T	G	0.378	0.463	1.020	0.0044	8.53E-06	1.028	0.0062	6.13E-06	3.52E-10	1.023	0.287	11.9	1.073	0.028	8.31E-03
2	58163178	rs2717026	A	G	0.623	0.537	0.980	0.0044	8.52E-06	0.972	0.0062	6.09E-06	3.52E-10	0.978	0.287	11.9	0.932	0.028	8.31E-03

2	58165595	rs2717029	T	G	0.378	0.465	1.020	0.0044	9.41E-06	1.028	0.0062	7.53E-06	4.46E-10	1.023	0.293	9.69	1.076	0.028	5.98E-03
2	58165694	rs2717030	T	C	0.622	0.542	0.981	0.0044	9.47E-06	0.972	0.0062	6.15E-06	4.20E-10	0.978	0.269	18.08	0.929	0.028	5.76E-03
2	58168831	rs2717038	A	G	0.623	0.538	0.980	0.0044	4.34E-06	0.972	0.0062	6.22E-06	1.61E-10	0.977	0.318	0	0.929	0.028	5.80E-03
2	58169166	rs2717039	T	G	0.377	0.460	1.021	0.0044	3.59E-06	1.028	0.0062	8.59E-06	1.61E-10	1.023	0.357	0	1.076	0.028	5.83E-03
2	58171964	rs2678898	T	C	0.620	0.537	0.981	0.0045	1.44E-05	0.973	0.0062	7.86E-06	1.09E-09	0.978	0.273	16.82	0.931	0.028	7.96E-03
2	58237405	rs4672228	T	C	0.612	0.502	0.982	0.0044	4.55E-05	0.974	0.0062	2.15E-05	6.62E-09	0.979	0.287	11.9	0.942	0.028	2.61E-02
2	58253423	rs13033536	A	G	0.373	0.503	1.019	0.0045	2.50E-05	1.026	0.0062	4.91E-05	8.00E-09	1.021	0.404	0	1.060	0.028	3.42E-02

Abbreviation

CHR, chromosome; POS, position; SNP, single nucleotide polymorphism; A1, allele 1; A2, allele 2; OR, odds ratio; SE, standard error.

Table S2. Overview of the differentially phosphorylated proteins between *Vrk2*$^{-/-}$ and *Vrk2*$^{+/+}$ mice.

Gene Name	Mol weight [kDa]	Sequence length	Position	AA	Sequence window	Vrk2$^{-/-}$ Intensity			Vrk2$^{+/+}$ Intensity			GroupB/ GroupA	*P* value
						GroupB-4	GroupB-5	GroupB-6	GroupA-1	GroupA-2	GroupA-3		
Ubr4	572.3	5180	226	S	ISPQALVEGENDEQSSPDQVSAAKTKSVFIA	245123.6		321869.2		35769.0	44943.4	7.02	0.024
Mical3	223.7	1993	1452	T	TLGSQSFNTSDSTMLTPPSSPPPPPPPNEEP	1897069.2	2149835.3	3375999.8	434864.9		343224.7	6.36	0.039
Mical3	223.7	1993	1456	S	QSFNTSDSTMLTPPSSPPPPPPPNEEPATLR	1897069.2	2149835.3	3375999.8	434864.9		343224.7	6.36	0.039
Psmd2	100.2	908	20	T	GRDKTPVQSQQPSATTPSGADEKSSGKERRD	582612.0		366467.8	186195.3	35688.2	38519.2	5.47	0.033
Smim13	10.0	88	70	S	NEQPSGSETEEDPSASPQKIRSARQRRPPVD	344611.8	384056.0	607126.4	59893.9	108826.4		5.28	0.043
Cdk14	53.0	469	134	S	SSPSSPTSPKFGKADSYEKLEKLGEGSYATV	1650301.3	1224611.6	1502772.7		512840.6	46691.9	5.22	0.016
Smim13	10.0	88	63	T	TGSQEGDNEQPSGSETEEDPSASPQKIRSAR	2014821.6	5607686.4	4571735.6	1331622.7	359781.5	649708.8	5.21	0.041
H1-4	22.0	219	2	S	______________MSETAPAAPAAPAPAEK	1804220.9	3021450.7	3334752.2	336845.3	737596.1		5.06	0.039
Prrt2	35.9	346	208	S	LQAGDGEEGPAPQPHSPPSTKTPPANGAPPR	2888253.9	3225053.8	3940701.9	1612988.9	367630.2	279290.2	4.45	0.0081
Cys1	15.5	145	116	S	KGNPEDSCASEAPGNSPKRPEGQSAISYDYS	206978.4	233562.6	300297.0	17080.0		118930.6	3.63	0.042
Jph1	71.9	660	461	T	PPSPKESPHFYRKGTTPPRSPESSPKQSHSP	344123.5	322684.3		79754.3	100679.7	110676.7	3.44	0.00047
Alad	36.0	330	215	S	FASCFYGPFRDAAQSSPAFGDRRCYQLPPGA	414454.5		312369.1	120107.1	167918.4	31777.6	3.41	0.028
Fgd1	106.4	960	98	S	PQHHRALRFSYHLEGSQPRPGLHQGNRILVK	11061955.4	13628403.7	10805647.1	653681.2	5306820.9	4578582.8	3.37	0.0081
Fgd1	106.4	960	92	S	LPCGPSPQHHRALRFSYHLEGSQPRPGLHQG	11061955.4	13628403.7	10805647.1	653681.2	3024990.1	7088324.9	3.30	0.017
Basp1	22.1	226	192	S	APSSKETPAASEAPSSAAKAPAPAAPAAAEP	844441.9	905234.7	1092598.0	98933.3	108039.3	665401.8	3.26	0.031
Zbtb20	81.0	741	432	S	AESSAQPNQLEPGASSPERSNESEMDNTVIT	1128340.8	1770693.3	1354292.1	279178.6	294124.0	746435.4	3.22	0.016
Uso1	107.0	959	940	S	SKLKDLGHPVEEEDESGDQEDDDDEIDDGDK	828678.6	1518852.4	1999312.9	250717.4	567449.2	541630.1	3.20	0.048
Pclo	550.8	5068	3506	S	GGSLGCQTETDPDTQSPPYMGATSPPKDKKR	2814740.2	3414376.8	3709463.4	2107181.1	666644.7	444630.1	3.09	0.019
Oxr1	95.9	866	346	S	TEESLSEDAFTESELSPIREELLSSEPRQEK	3214179.3	1817840.1	2622364.6	431046.3	846347.2	1236693.9	3.04	0.021
Sgip1	86.1	806	335	T	PEHVTPELTPREQVVTPPAASDIPADSPAPA	5026362.1	6409429.8	8240542.2	1611336.9	2657304.3	2570795.9	2.88	0.012
Slc38a3	55.6	505	53	S	TQHCGEGKGFLQKSPSKEPHFTDFEGKTSFG	172958.2	269147.8		82968.3	94915.6	56064.8	2.83	0.035
Lsm14a	50.5	462	183	S	QDTRSVKPQLAQGRSSPQLDPLRKSPTMEQA		1219922.7	1189962.5	480459.4	402490.1	394896.5	2.83	0.00023
Clip4	75.8	704	580	S	SNKQNHSYPGFRRSFSTTSASSQKEINRRNA	152114.6	126908.2	106525.8	60767.0	46449.3	32530.3	2.76	0.0061
Map1a	300.1	2776	895	S	EATQGLDYVPSAGTISPTSSLEEDKGFKSPP	14802999.2	13587497.7	19453709.0	4397554.1	5489777.0	8101646.5	2.66	0.0090
Cd34	41.0	382	343	S	TENGGGQGYSSGPGASPETQGKANVTRGAQE	142186.1	147633.6			65850.9	43419.4	2.65	0.016
Hsph1	96.4	858	558	S	PQVQTDGQQTSQSPPSPELTSEESKTPDADK	3158099.3	4209161.7	4023592.3	1980523.0	1242459.7	1185503.7	2.58	0.0049
Arhgap35	170.4	1499	975	S	ACSTTEEVFNSPRAGSPLCNSNLQDSEEDVE	1789377.0	1743053.5	2548831.9	987802.3	495382.4	931398.5	2.52	0.016
Syngap1	148.2	1340	892	S	LTAALGLRPAPAGRLSQGSGSSITAAGMRLS	695075.4	620605.8	1018452.2	327547.5	417261.8	199844.3	2.47	0.028
Wdr44	101.6	915	27	S	DAPEDVHLGTGYPVGSPGKVGLLSFKEAENT	852692.3	636207.0	619934.1		285712.9	294617.2	2.42	0.024
Slc20a1	74.2	681	269	S	VCPRMKRKIEREVKSSPSESPLMEKKSNLKE	1500363.2	1462555.2	1596742.2	659112.6	598235.9		2.42	0.00055
Nckipsd	78.6	714	260	S	HTTVSQPQPSPSKAPSPEPPTEEVAAETNST	4997677.8	4677095.4	5386550.5	1175585.3	3147538.8	2261738.3	2.29	0.0096
Ttyh3	57.7	524	437	S	DGEEETAPGPRQAHDSLYRVHMPSLYSCGSS	162441.8	187179.3	219747.0	71433.3	91581.6	88088.6	2.27	0.0039
Rmdn3	52.0	470	212	S	EDEVSCETVRMGRKDSLDLDVEAASSPAAAA	229016.5	262403.2		91185.4	104192.4	134893.7	2.23	0.0074
Camkv	54.8	512	436	S	ITPATDGSVTPATDRSATPATDGRATPATEE	988391.6	919456.6	1385483.3	338784.7	796762.9	360103.6	2.20	0.045
Ank2	426.3	3898	2206	S	EESYKHEGLAETPETSPESLSFSPKKSEEQI	232664.5	269324.2		110820.1	94274.0	145354.6	2.15	0.011
Jph3	81.2	744	420	S	AQEEARIARITAKEFSPSFQHRENGLEYQRP	1343248.2	1588710.6	2067568.5	683329.1	911273.1	790288.9	2.10	0.017
Rtn3	103.9	964	31	S	SSSGAEPSALGGGGGSPGACPALGAKSCGSS	13114235.0	15490494.0	18949774.3	11552339.3	3577244.7	8176381.2	2.04	0.048
Numbl	64.1	604	263	S	AAPAVAPGPAQPGHVSPTPATTSPGEKGEAG		2114145.5	2371076.0	1191822.7	711214.9	1394736.6	2.04	0.026
Sos1	150.9	1319	1120	S	FDSDHSASPFHSRSASVSSISLSKGTDEVPV	50181.8	53106.7		29433.6	21276.2		2.04	0.026
Cntnap2	148.2	1332	1304	S	RHKGTYHTNEAKGAESAESADAAIMNNDPNF	904017.3	1028639.7	884657.6	286081.0	628078.2	486043.1	2.01	0.012
Cntnap2	148.2	1332	1307	S	GTYHTNEAKGAESAESADAAIMNNDPNFTET	1183936.6	1340598.5	1231618.4	424899.3	824494.5	618678.3	2.01	0.0072
Rtn4	126.6	1162	953	S	VKPKVLTKEAEEKLPSDTEKEDRSLTAVLSA	4171989.3	2696642.2	3148040.6	1085250.9	1760471.1	2145914.7	2.01	0.035
Ppp1r7	41.3	361	12	S	____MAAERGAGQQQSQEMMEVDRRVESEES	73112.1	66487.1	43578.7	102469.3	129740.6	135753.6	0.50	0.011
Tagln3	22.5	199	163	S	PSWFHRKAQQNRRGFSEEQLRQGQNVIGLQM	212729.6	260794.9	100513.2	328307.7	427544.7	400820.5	0.50	0.026
Snx2	58.5	519	185	S	LSMFSKSEFSVKRRFSDFLGLHSKLASKYLH	33669.9	31624.6	17529.5	45172.1	61525.8	63272.6	0.49	0.019

Psd3	114.7	1037	719	S	EGGDFSKDLLKALYNSIKNEKLEWAVDDEEK	18991.1		37005.3	59205.5	57044.8	56422.6	0.49	0.022
Nostrin	57.7	506	479	S	IVTVHEKKEEGWWFGSLKGKRGHFPAAYVEE		34678.5	51540.1		91961.4	85657.6	0.49	0.037
Pkp4	131.6	1190	280	S	LPAQRAASPYSQRPASPTAVRRVGSVTSRQT	146512.4	632400.2	538352.4	829705.6	874223.8	1011746.0	0.49	0.042
Shank1	226.3	2167	615	S	VGWFPSDCLEEVANRSQEGRQESRSDKAKRL	480547.2	610964.8	409141.4	787380.3	1292477.2	1022519.6	0.48	0.027
Slc4a3	135.4	1227	175	S	PPQKAKFSIGSDEDDSPGLPVKAPCAKALPS	123574.5	63573.3	59167.2		173444.4	166389.7	0.48	0.047
Ctnnal1	81.5	731	373	S	QLLSVWMQTQSRKTKSAAEELELTVLKISHS	24708.7	22760.7		37578.4	52394.9	57707.5	0.48	0.047
Pgm2	68.7	620	173	S	TVSHLKLCAGIMITASHNPKQDNGYKVYWDN	88016.1	88006.0	71551.7	186478.2	141691.0	193165.1	0.47	0.0059
Nsfl1c	40.7	370	176	S	PEEESAYVAGERRRHSGQDVHVVLKLWKTGF	873427.6	984514.7	1599931.0	2613289.8	2202723.7	2479200.8	0.47	0.0075
Sgip1	86.1	806	528	S	LSAATTPTVGSSRGPSPLTMGAQDTLPVAAA	29021.8		18074.2	50891.4		49553.2	0.47	0.040
Sgip1	86.1	806	484	S	LPPGKPGVGDVSRPFSPPIHSSSPPPIAPLA		894917.3	1031806.5	2289761.3	1862126.3		0.46	0.038
Pip4p1	30.0	284	169	S	CKRIINLGPVHPGPLSPEPQPMGVRVICGHC	175280.7	233417.3	99924.9	399433.1	254502.5	451932.0	0.46	0.048
Nrbp2	57.3	499	207	S	VWYRIFSNALPDDLRSPIRAEREELRNLHFF	150739.0	62212.1	196438.2	321485.1	284013.0	288937.8	0.46	0.017
Nsfl1c	40.7	370	140	S	GAKEHGAVAVERVTKSPGETSKPRPFAGGGY	52740.3	24655.3		91600.6	85653.3	76888.5	0.46	0.030
Kcnab1	44.7	401	60	S	IIARSLGTFTPQHHISLKESTAKQTGMKYRN	35074.0	46221.5	9100.5	61232.4	62730.9	76977.5	0.45	0.038
Cldn11	22.1	207	198	S	QSFGENRFYYSSGSSSPTHAKSAHV______	5954299.3	6895677.2	8602328.4	12374248.9	17050066.5	18672669.8	0.45	0.012
Syn1	74.1	706	427	S	NKMTQALPRQPQRDASPGRGSHSQSSSPGAL	1138603.1	703095.6	590521.2	1770978.9	1649494.6	2064983.5	0.44	0.0080
Ampd2	92.0	798	53	S	MDGKCKEIAEELFSRSLAESELRSAPYEFPE	23915.8	29044.8	20177.9	44554.8	67047.0		0.44	0.040
Brsk2	81.7	735	424	S	QRSRSISGASSGLSTSPLSSPRVTPHPSPRG	916512.1	829929.0	738549.6	1716278.2	2355112.2	1702148.6	0.43	0.0078
Syt3	63.3	587	382	S	FSVPLAELAQRKLHFSVYDFDRFSRHDLIGQ	173047.9	99134.9	42841.7	242399.3	202545.9	293544.1	0.43	0.037
Mbp	27.2	250	206	S	DSHTRTTHYGSLPQKSQHGRTQDENPVVHFF	86754.0	127560.6	53377.1	162454.1	273193.9	198032.8	0.42	0.035
Rbm12	102.8	992	424	S	PRSKSPSGQKRSRSRSPHEAGFCVYLKGLPF	72412.2	70752.5	103225.4	186888.0	207697.0	200562.8	0.41	0.00068
Nefl	61.5	543	13	S	___MSSFGYDPYFSTSYKRRYVETPRVHISS	20764.4	31176.9	12030.0		48591.5	56177.0	0.41	0.027
Map1a	300.1	2776	1797	T	PSAPGQESPVPDTKSTPPTRNEPTTPSWLAE	2892636.8	1890548.6	2029504.8	4759884.9	6055727.2	6150966.6	0.40	0.0035
Kcnj3	56.6	501	385	S	PLIAPAITNSKERHNSVECLDGLDDISTKLP	34738.9	52832.9	58960.7	108798.4	133350.5	124119.9	0.40	0.0020
Map1b	270.3	2464	1775	S	SLASEKVQSLEGEKLSPKSDISPLTPRESSP	719329.3	849612.5	255646.3	1346592.6	1957697.2	1270247.4	0.40	0.032
Grin2b	166.0	1482	930	S	NGSPQSALDFIRRESSVYDISEHRRSFTHSD	111053.4	174429.9	251608.8	448180.8	588379.7	318024.1	0.40	0.036
Phyhipl	42.3	375	12	S	____MEVPRLDHALSSPSSPCEEIKNLSLEA	308333.7	187283.0	281176.9	684010.4	779128.3	521706.0	0.39	0.0086
Git1	85.3	770	419	S	EPLPSAGATRNNRARSMDSSDLSDGAVTLQE	158474.4	64470.4	94883.4	228911.6		325627.6	0.38	0.043
Mical3	223.7	1993	1406	S	KSAGDQPPLLTPKSPSDKELRSSQEERRDLS	26995.4	59668.7		118066.2		118424.9	0.37	0.044
Mical3	223.7	1993	1404	S	LPKSAGDQPPLLTPKSPSDKELRSSQEERRD	26995.4	59668.7		118066.2		118424.9	0.37	0.044
Nipbl	315.5	2798	2666	S	GSPKNNTAADTEDEESDGEDRGGGTSGSLRR		54239.0	37652.6	126375.8	124694.5		0.37	0.011
Relch	134.6	1216	56	S	LGAGVGLDPGSAGSLSPQDPMALGSSARPGL	243548.8	164864.3	160686.1		546635.6	530629.7	0.35	0.0022
Scn2a	227.9	2006	488	S	DFSGAGGIGVFSESSSVASKLSSKSEKELKN	80585.6	52010.6	143058.6		278441.4	244093.8	0.35	0.020
Hnrnpm	77.6	729	452	S	GLVMDRMGSVERMGSSIERMGPLGLDHMASS	38537.2		15540.4	74684.1	84180.0	73869.3	0.35	0.013
Phyhipl	42.3	375	11	S	_____MEVPRLDHALSSPSSPCEEIKNLSLE	308333.7	187283.0	192510.1	684010.4	779128.3	521706.0	0.35	0.0070
Ak5	63.3	562	181	S	LLRKKIHSASSNRKWSLIAKIITNGELAPQE	11449.8		9807.1	28060.0	33399.3		0.35	0.019
Rims1	163.2	1463	1448	S	SSLVDPTLTPLTRRASQSSLESSSGPPCIRS	108700.7	577385.9		1091474.6	914079.2	1048769.9	0.34	0.037
Rab3c	25.9	227	211	S	SESLETDPAITAAKQSTRLKETPPPPQPNCG		24271.8	19814.8	65913.4		66408.7	0.33	0.0026
Syn1	74.1	706	438	S	QRDASPGRGSHSQSSSPGALTLGRQTSQQPA	2015020.8	467526.5	581879.6	4020810.1	3543062.8	2262549.7	0.31	0.036
Itpr2	307.5	2701	937	S	HGVGEMMTQMVLSRGSIFPVSVPDAQPIVHP	90452.7	16823.3	55776.5	158208.7	160769.6	205764.7	0.31	0.010
C2cd5	111.7	1016	659	S	PIPEPRQRSRLLRSQSESSDEVTELDLSHGK	30863.7	24627.5			100404.2	91267.1	0.29	0.0065
Tmem63b	94.8	832	114	S	HGDSHDRYERLTSVSSSVDFDQRDNGFCSWL	26126.8	41402.8	20591.9	97248.2		119992.8	0.27	0.0065
Ptdss1	55.6	473	442	S	EISWHHGKGSKGSEDSPPKHSSNHESHSSRR	32593.9	36182.0			139714.1	115076.7	0.27	0.017
Ncam1	119.4	1115	788	T	ESKEPIVEVRTEEERTPNHDGGKHTEPNETT	1445268.8	216252.4		3617220.8	3428369.0	2512572.3	0.26	0.034
Pclo	550.8	5068	1563	S	RKSSTSFDDDAGRRHSWHDEDDETFDESPEL	166528.7	168688.3	32631.9	443573.4	778470.8	332320.1	0.24	0.049
Dbn1	77.3	706	387	S	SHRRMAPTPIPTRSPSDSSTASTPIAEQIER		360926.6	378977.9	1524847.9	1704395.8		0.23	0.0052
Rims1	163.2	1463	1110	S	IHKDQYRSCDNASAKSSDSDVSDVSAISRAS		54826.8	38622.0	232432.4	186766.9		0.22	0.021
Fip1l1	65.0	581	479	S	RERERTRERERERDHSPTPSVFNSDEERYRY	40743.9	52801.9	45797.7	273709.1	253559.0	170346.1	0.20	0.0043
Clec2l	23.7	211	29	S	PPPAARPAPAAPRPRSPAEAEARGPEGLLRR	42608.6	138683.7		378231.7	411084.1	584103.7	0.20	0.027
Cdkn2aip	59.7	563	169	S	KSAKTDRSAAQQENSSPSRGSSTKSESGGTS		63750.0	13676.0	176135.5	168537.9	242636.3	0.20	0.022

Dlg2	94.9	852	574	S	GLSFKYGDILHVINASDDEWWQARRVTLDGD		4139.4	2644.1	15236.4		19462.1	0.20	0.025
Slc9a3r1	38.6	355	294	S	ALARSASSDTSEELNSQDSPKRQVSTEPSST	29261.2	83065.7		337790.7	423822.5	368366.2	0.15	0.0035
Slc4a4	121.5	1079	68	S	EKKEKERISENYSDKSDVENADESSSSILKP	85626.4	57308.6			1196166.8	1452625.5	0.05	0.010

References

1. Wray NR, Ripke S, Mattheisen M, Trzaskowski M, Byrne EM, Abdellaoui A, Adams MJ, Agerbo E, Air TM, Andlauer TMF *et al*: **Genome-wide association analyses identify 44 risk variants and refine the genetic architecture of major depression**. *Nat Genet* 2018, **50**(5):668-681.
2. Howard DM, Adams MJ, Shirali M, Clarke TK, Marioni RE, Davies G, Coleman JRI, Alloza C, Shen X, Barbu MC *et al*: **Genome-wide association study of depression phenotypes in UK Biobank identifies variants in excitatory synaptic pathways**. *Nat Commun* 2018, **9**(1):1470.
3. Hyde CL, Nagle MW, Tian C, Chen X, Paciga SA, Wendland JR, Tung JY, Hinds DA, Perlis RH, Winslow AR: **Identification of 15 genetic loci associated with risk of major depression in individuals of European descent**. *Nat Genet* 2016, **48**(9):1031-1036.
4. Converge consortium: **Sparse whole-genome sequencing identifies two loci for major depressive disorder**. *Nature* 2015, **523**(7562):588-591.
