# Supplementary material for: Reduced Vrk2 expression is associated with higher risk of depression in humans and mediates depressive-like behaviors in mice
Source: BMC Med. 2023 Jul 14;21:256. doi: 10.1186/s12916-023-02945-0 (PMC10349461; doi:10.1186/s12916-023-02945-0)
Supplement: Supplementary file 2 — Additional file 2. Figure S1. Flow chart ofthe genetic analyses. Figure S2. The linkage disequilibrium (LD) maps of theVRK2 SNPs in European and Han Chinese individuals from 1000 GenomesProject. Figure S3. Functional prediction of the 47 SNPs usingHaploReg v.4.2. Figure S4. Functional prediction of the 47 SNPs usingGWAVA. Figure S5. Expression quantitative trait loci (eQTL)analyses of rs2678907 with FANCL mRNAin the DLPFC tissues from CommonMind Consortium dataset. Figure S6. A. Knockdown efficiency of Vrk2 in thewild-type C57BL/6J male mice injected with Vrk2-shRNA AAV in the ventralhippocampus. B. Knockout efficiency of Vrk2 in Vrk2-/- mice. C. Overexpression efficiency of Vrk2 in the ventralhippocampus of Vrk2-/- mice injected with Vrk2 overexpressingAAV. Figure S7. Knockdown efficiency of Vrk2 mediated byshRNAs. Figure S8. Behavioral analyses of Vrk2+/+and Vrk2-/- mice after chronic restraint stress (CRS). FigureS9. Behavioral analyses of Vrk2+/+, Vrk2+/-and Vrk2-/- mice. [file 12916_2023_2945_MOESM2_ESM.pdf]

Figure S1. Flow chart of the genetic analyses.

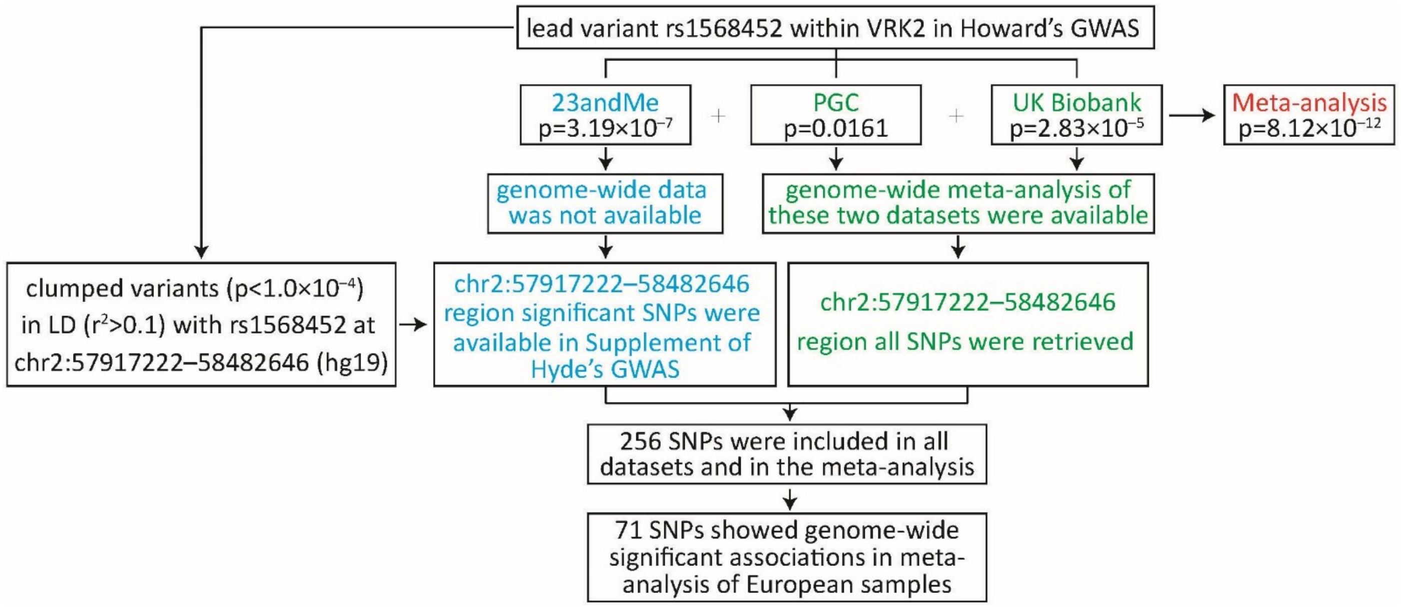

Figure S2. The linkage disequilibrium (LD) maps of the *VRK2* SNPs in European and Han Chinese individuals from 1000 Genomes Project [1]. The figure was generated using the Haploview software [2], and the LD of the tested SNPs was calculated using r^2 algorithm implemented in the Haploview program.

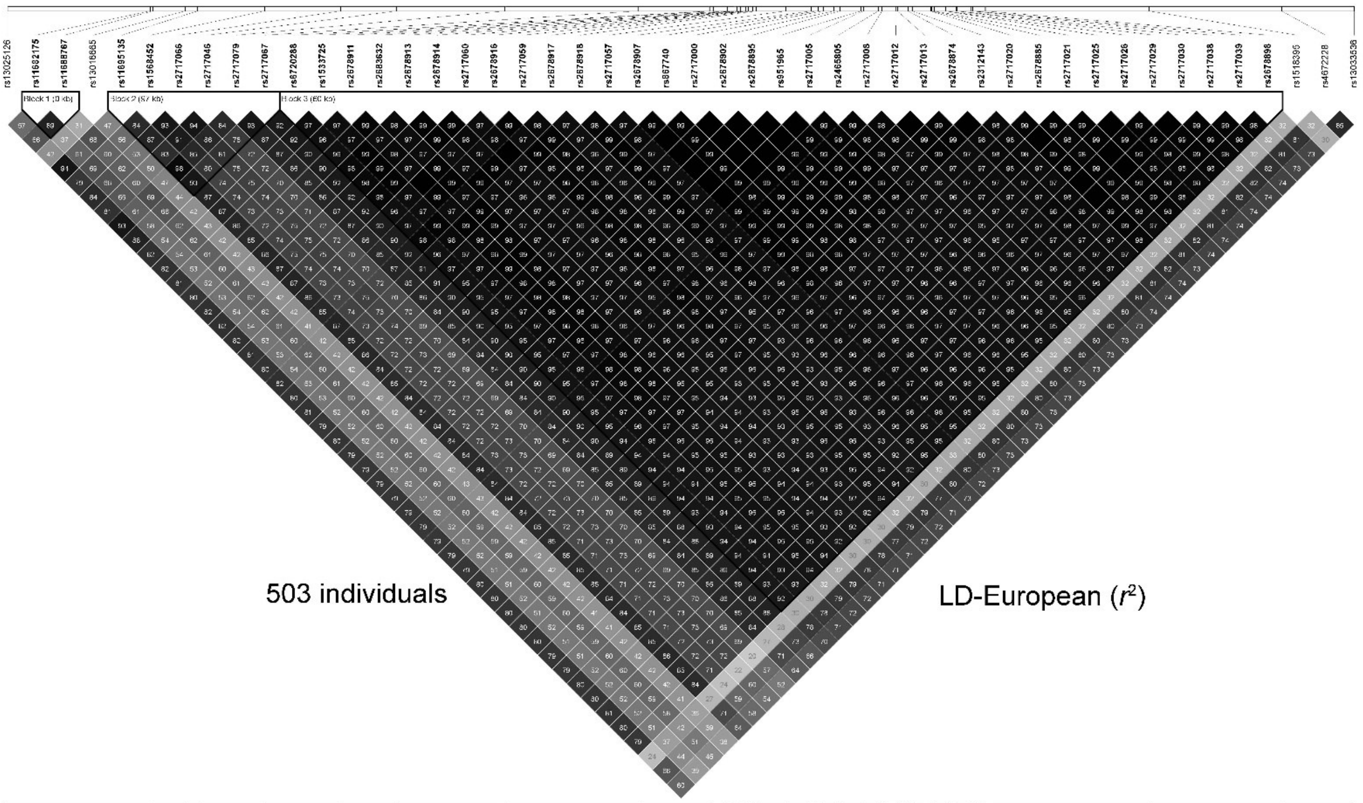

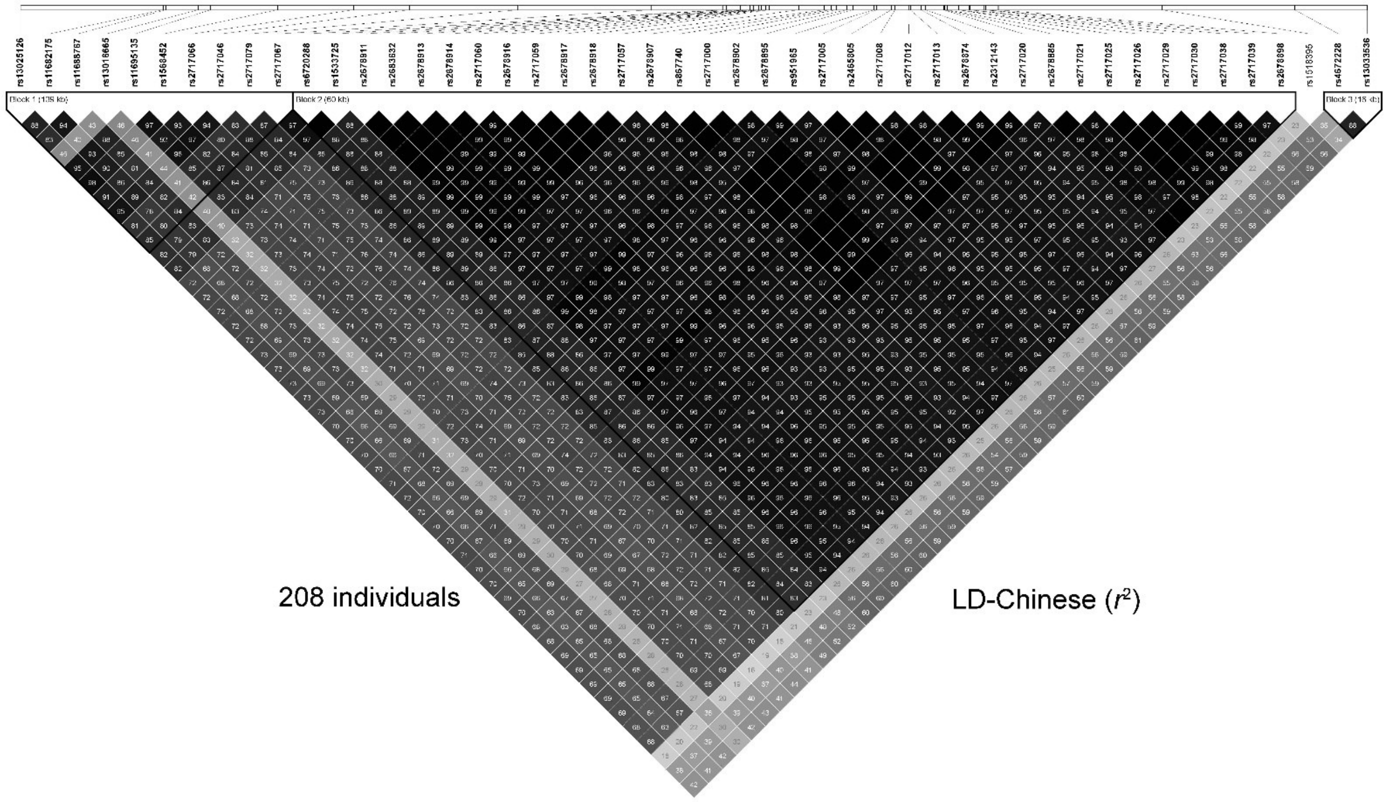

Figure S3. Functional prediction of the 47 SNPs using HaploReg v.4.2 [3].

variant	Ref	Alt	AFR freq	AMR freq	ASN freq	EUR freq	SiPhy cons	Promoter histone marks	Enhancer histone marks	DNAse	Proteins bound	Motifs changed
rs13025126	G	A	0.03	0.41	0.41	0.38				MUS		5 altered motifs
rs11682175	T	C	0.20	0.47	0.44	0.44		16 tissues	12 tissues	4 tissues		8 altered motifs
rs11688767	A	T	0.20	0.48	0.46	0.47			5 tissues			9 altered motifs
rs13016665	C	A	0.35	0.50	0.58	0.44						7 altered motifs
rs11695135	A	C	0.03	0.40	0.42	0.39						EWSR1-FLI1,GATA,PU.1
rs1568452	C	T	0.14	0.41	0.41	0.36						
rs2717066	C	A	0.04	0.38	0.41	0.36				PLCNT	YY1	5 altered motifs
rs2717046	C	T	0.12	0.40	0.41	0.37			LNG			NF-kappaB,STAT
rs2717079	T	A	0.13	0.41	0.45	0.39						Arid5a,YY1
rs2717067	G	C	0.05	0.40	0.43	0.39			MUS, LNG			Foxc1
rs6720288	A	G	0.34	0.42	0.43	0.38			FAT, LNG, BLD			Dobox4
rs1533725	T	G	0.14	0.40	0.42	0.39			LNG, BLD			
rs2678911	G	C	0.05	0.39	0.46	0.39			BLD			6 altered motifs
rs2683632	T	C	0.34	0.42	0.46	0.39						4 altered motifs
rs2678913	T	C	0.34	0.42	0.46	0.39			FAT, BRN			Arid5b,Zbtb12
rs2678914	C	G	0.14	0.40	0.46	0.39			8 tissues	8 tissues	4 bound proteins	Hdx,Homez,Pou3f1
rs2717060	A	G	0.25	0.41	0.46	0.39			BRN, GI, BLD	BRN,BRN		EWSR1-FLI1,Ik-2,NF-AT1
rs2678916	G	T	0.05	0.40	0.46	0.39			BRN, GI, BLD			7 altered motifs
rs2717059	C	G	0.35	0.42	0.46	0.39			BRN, GI, BLD			4 altered motifs
rs2678917	T	A	0.25	0.41	0.46	0.39		BLD	5 tissues	BRN,BLD		7 altered motifs
rs2678918	C	T	0.05	0.40	0.46	0.39			5 tissues	BRN,BRN		CDP,Hbp1,TCF11::MafG
rs2717057	C	T	0.34	0.41	0.46	0.38						Mef2
rs2678907	A	G	0.14	0.39	0.46	0.38			4 tissues	42 tissues	8 bound proteins	NR4A
rs867740	T	C	0.05	0.40	0.46	0.39		BRN	FAT, BLD, LNG			Irf
rs2717000	C	G	0.14	0.39	0.46	0.39		BRN	BLD	6 tissues	4 bound proteins	Pou2f2,STAT
rs2678902	G	A	0.14	0.39	0.46	0.39			ESDR, BRN			Maf
rs2678895	A	T	0.05	0.39	0.46	0.38			FAT, BRN			Ets,GATA,HDAC2
rs951965	C	A	0.14	0.39	0.46	0.39						ERalpha-a,Pbx3
rs2717005	A	G	0.06	0.39	0.46	0.39						7 altered motifs
rs2465805	C	T	0.25	0.39	0.46	0.38			BRN			HDAC2,TCF11::MafG
rs2717008	T	C	0.14	0.40	0.46	0.38			ESDR, BRN	BRN,BRN		
rs2717012	C	A	0.05	0.38	0.46	0.39						Arid5a,Pou2f2,Pou3f3
rs2717013	G	A	0.10	0.39	0.46	0.39						
rs2678874	T	A	0.10	0.39	0.46	0.38						4 altered motifs
rs2312143	G	A	0.10	0.39	0.46	0.39						CDP,Foxa,Pou2f2
rs2717020	T	C	0.10	0.39	0.46	0.38						4 altered motifs
rs2678885	T	G	0.05	0.39	0.47	0.38						9 altered motifs
rs2717021	G	A	0.10	0.39	0.46	0.38						5 altered motifs
rs2717025	G	T	0.10	0.39	0.46	0.38						Myf,TATA,ZEB1
rs2717026	A	G	0.10	0.39	0.46	0.38						
rs2717029	G	T	0.05	0.39	0.47	0.38						Irx
rs2717030	T	C	0.10	0.39	0.46	0.38						CCNT2
rs2717038	A	G	0.12	0.40	0.46	0.38			BRN			5 altered motifs
rs2717039	G	T	0.05	0.39	0.46	0.38			ESC, IPSC, BRN	ESDR		Rhox11,Zbtb3
rs2678898	T	C	0.11	0.39	0.46	0.38						EWSR1-FLI1,HDAC2,VDR
rs4672228	T	C	0.07	0.41	0.50	0.40		ESDR	ESDR, LNG			CDP
rs13033536	G	A	0.08	0.41	0.50	0.40						5 altered motifs

Figure S4. Functional prediction of the 47 SNPs using GWAVA [4].

SNP	CHR	Position	Region score	TSS score	Unmatched score	TSS distance	CTCF	DNase	FAIRE
rs13025126	2	57,956,088	0.28	0.16	0.02	-178697	-	-	-
rs11682175	2	57,987,593	0.27	0.39	0.41	-147192	-	-	5
rs11688767	2	57,988,194	0.33	0.24	0.2	-146591	-	-	-
rs13016665	2	57,995,348	0.31	0.09	0	-139437	-	-	-
rs11695135	2	57,998,040	0.32	0.19	0.01	-136745	-	-	-
rs1568452	2	58,012,833	0.45	0.28	0.02	-121952	-	-	-
rs2717066	2	58,029,645	0.76	0.71	0.5	-105140	-	1	-
rs2717046	2	58,041,936	0.38	0.21	0.04	-92849	-	-	-
rs2717079	2	58,065,936	0.58	0.6	0.21	-68849	-	-	-
rs2717067	2	58,095,252	0.36	0.17	0.04	-39533	-	-	-
rs6720288	2	58,111,064	0.27	0.38	0.21	-23721	-	-	-
rs1533725	2	58,111,922	0.45	0.75	0.42	-22863	-	-	-
rs2678911	2	58,115,072	0.41	0.51	0.3	-19713	-	-	-
rs2683632	2	58,116,640	0.23	0.19	0.03	-18145	-	-	-
rs2678913	2	58,117,095	0.22	0.26	0.07	-17690	-	-	-
rs2678914	2	58,117,926	0.44	0.56	0.27	-16859	-	5	3
rs2717060	2	58,119,122	0.37	0.49	0.26	-15663	-	-	1
rs2678916	2	58,119,582	0.3	0.43	0.3	-15203	-	-	-
rs2717059	2	58,119,657	0.21	0.5	0.15	-15128	-	-	-
rs2678917	2	58,120,553	0.37	0.57	0.38	-14232	-	-	2
rs2678918	2	58,121,391	0.57	0.56	0.36	-13394	-	-	-
rs2717057	2	58,127,907	0.22	0.28	0.12	-6878	-	-	-
rs2678907	2	58,133,522	0.5	0.56	0.47	-1263	57	104	15
rs867740	2	58,135,023	0.19	0.25	0.42	231	-	1	2
rs2717000	2	58,136,220	0.33	0.29	0.36	1428	-	1	2
rs2678902	2	58,138,581	0.27	0.27	0.15	3789	-	-	-
rs2678895	2	58,139,906	0.19	0.13	0.14	5114	-	2	-
rs951965	2	58,144,542	0.31	0.04	0.05	9750	-	-	-
rs2717005	2	58,145,060	0.39	0.11	0.02	10268	-	-	-
rs2465805	2	58,148,349	0.25	0.1	0	13557	-	-	-
rs2717008	2	58,149,158	0.41	0.23	0.13	14366	-	-	1
rs2717012	2	58,152,280	0.29	0.04	0	17488	-	-	-
rs2717013	2	58,152,537	0.23	0.01	0	17745	-	-	-
rs2678874	2	58,154,915	0.34	0.03	0	20123	-	-	-
rs2312143	2	58,155,829	0.26	0.02	0	21037	-	-	-
rs2717020	2	58,159,852	0.3	0.04	0.02	25060	-	-	1
rs2678885	2	58,160,138	0.27	0.02	0.02	25346	-	-	-
rs2717021	2	58,160,605	0.23	0.03	0	25813	-	-	-
rs2717025	2	58,163,146	0.28	0.09	0.02	28354	-	-	-
rs2717026	2	58,163,178	0.17	0.04	0.02	28386	-	-	-
rs2717029	2	58,165,595	0.21	0.04	0.02	30803	-	-	-
rs2717030	2	58,165,694	0.25	0.09	0.04	30902	-	-	-
rs2717038	2	58,168,831	0.37	0.37	0.2	34039	-	-	-
rs2717039	2	58,169,166	0.4	0.34	0.22	34374	-	-	-
rs2678898	2	58,171,964	0.25	0.28	0.05	37172	-	-	-
rs4672228	2	58,237,405	0.2	0.21	0.02	-36246	-	-	-
rs13033536	2	58,253,423	0.34	0.29	0.23	-20228	-	-	-

Abbreviation:

SNP, single nucleotide polymorphism; CHR, chromosome; TSS, transcriptional start site.

Figure S5. Expression quantitative trait loci (eQTL) analyses of rs2678907 with *FANCL* mRNA in the DLPFC tissues from CommonMind Consortium dataset [5].

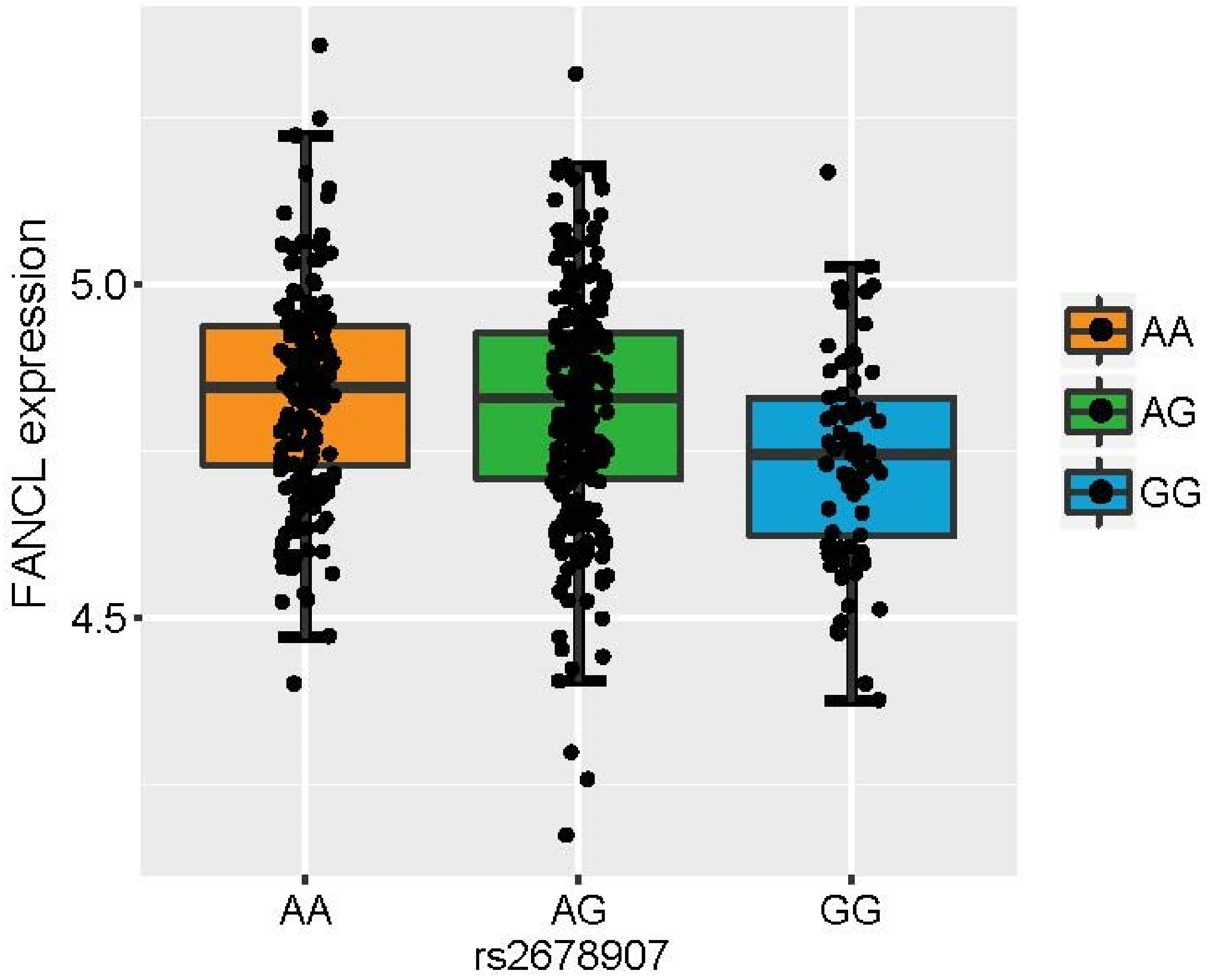

Figure S6. A. Knockdown efficiency of Vrk2 in the wild-type C57BL/6J male mice injected with *Vrk2*-shRNA AAV in the ventral hippocampus. B. Knockout efficiency of Vrk2 in *Vrk2*$^{-/-}$ mice. C. Overexpression efficiency of Vrk2 in the ventral hippocampus of *Vrk2*$^{-/-}$ mice injected with Vrk2 overexpressing AAV.

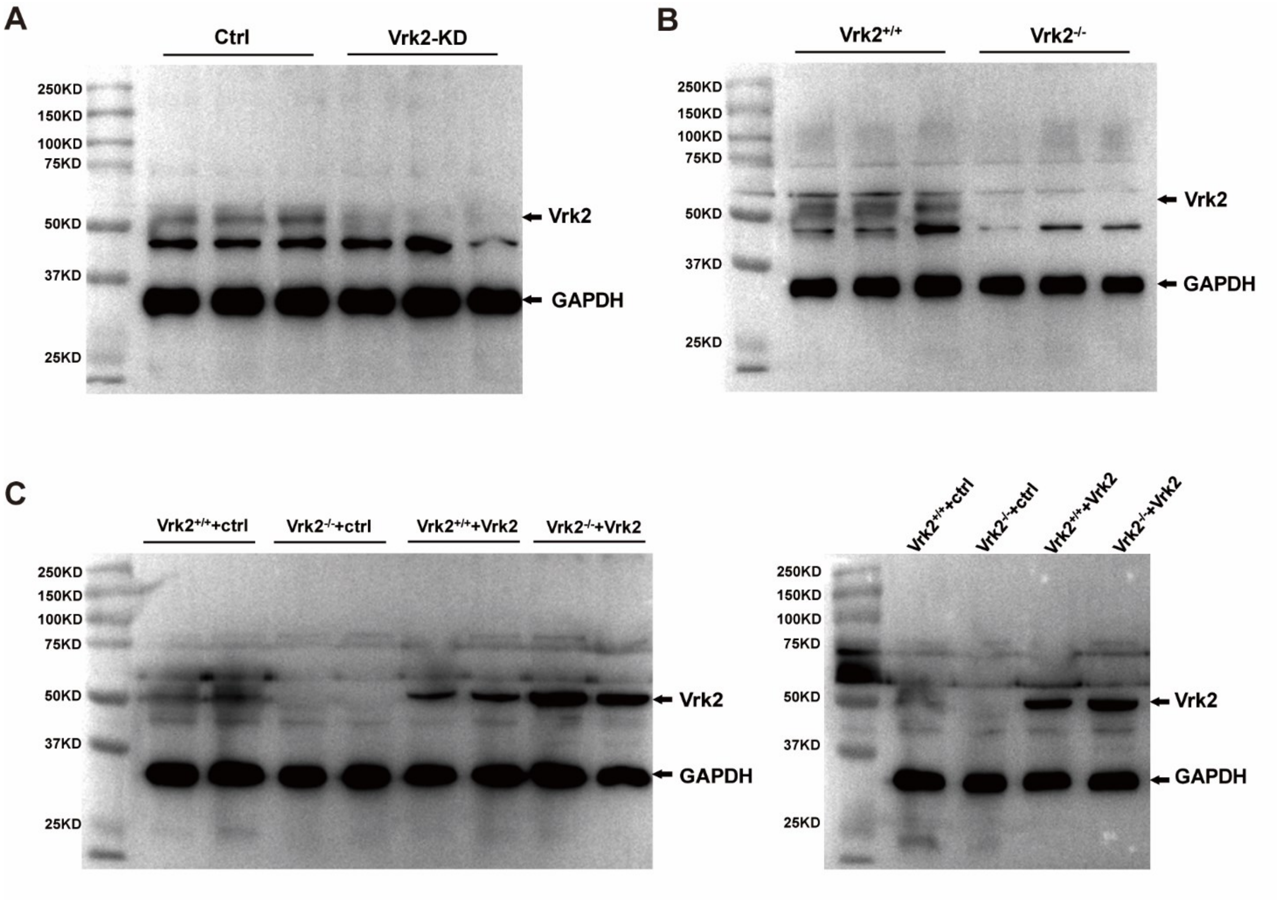

Figure S7. Knockdown efficiency of Vrk2 mediated by shRNAs. A. ***VRK2*** **mRNA knockdown efficiencies were measured in NIH3T3 cells infected with AAV that contained** ***VRK2*****-shRNA.** *Vrk2*-sh1 ($P = 0.0043$) and *Vrk2*-sh5 ($P = 0.0035$) had the best knockdown efficiencies among the group. $n = 2$ per group. **B. The AAV containing** ***VRK2*****-shRNA was injected into the ventral hippocampus of wild-type C57BL/6J male mice, and the knockdown efficiencies of Vrk2 were detected by extracting the protein after stable expression of the virus.** The results indicated that *Vrk2*-sh1 ($P = 0.0468$) had a higher protein level knockdown efficiency. $n = 3$ per group. One-way ANOVA for A-B. Error bars represent mean ± standard deviation (SD).

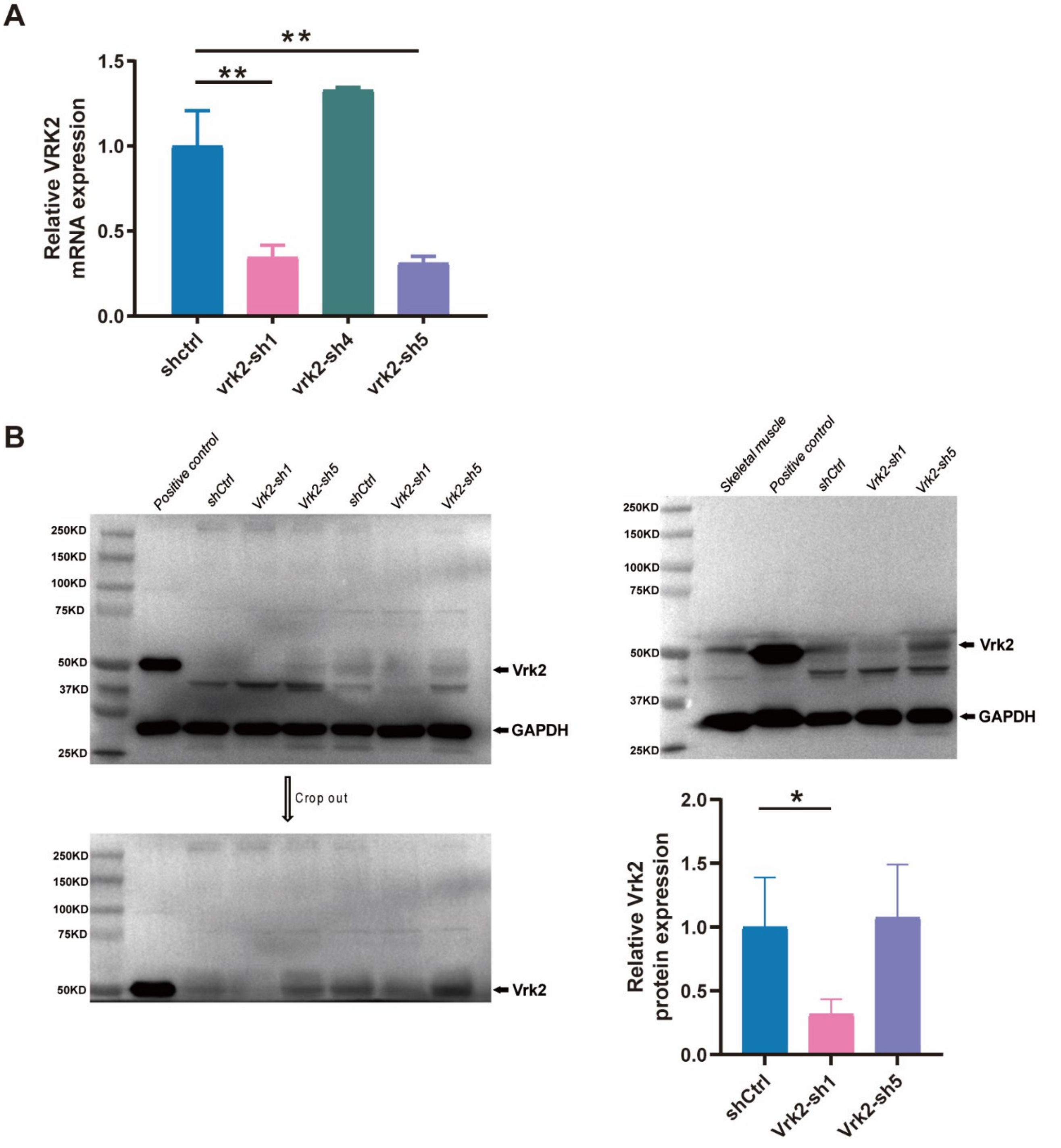

Figure S8. Behavioral analyses of *Vrk2*$^{+/+}$ and *Vrk2*$^{-/-}$ mice after chronic restraint stress (CRS). A Open field test. No difference was seen in the total distance (P = 0.566) and central exploration time (P = 0.218) between *Vrk2*$^{+/+}$ and *Vrk2*$^{-/-}$ mice. *Vrk2*$^{+/+}$, n = 6; *Vrk2*$^{-/-}$, n = 9. **B Rotarod test.** *Vrk2*$^{+/+}$, n = 6; *Vrk2*$^{-/-}$, n = 9. P = 0.648. **C Sucrose preference test.** *Vrk2*$^{+/+}$, n = 6; *Vrk2*$^{-/-}$, n = 8. P= 0.832. **D Tail suspension test.** There was significant reduction in curling time (P = 0.0082) in *Vrk2*$^{-/-}$ mice compared with *Vrk2*$^{+/+}$ mice, but no difference in immobility time (P = 0.221). *Vrk2*$^{+/+}$, n = 5; *Vrk2*$^{-/-}$, n = 8. * P < 0.05, ** P < 0.01, *** P < 0.001 (two-tailed t-test). Error bars represent mean ± standard deviation (SD).

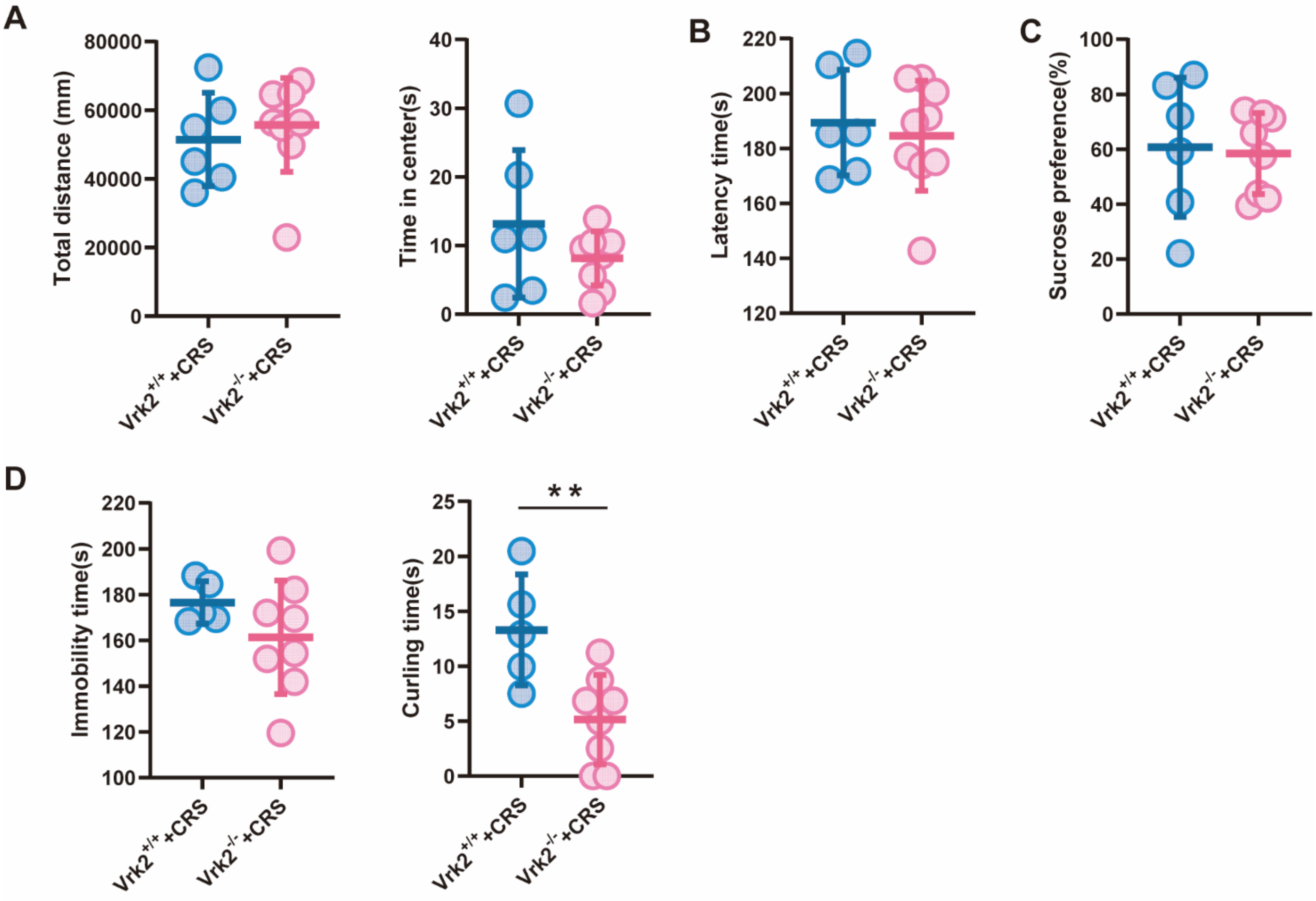

Note: Chronic restraint stress (CRS) in *Vrk2*$^{-/-}$ and *Vrk2*$^{+/+}$ mice was performed as previously reported [6]. In brief, the mice were placed in conical tubes (50 mL) for six hours per day (10:00 a.m. to 16:00 p.m.) for a total of 14 days. To allow the mice to breathe normally, the conical tubes included holes in the side walls and bottom. The mice were unable to move their bodies freely during the duration of restraint stress and were denied access to food and drink.

Figure S9. Behavioral analyses of *Vrk2*$^{+/+}$, *Vrk2*$^{+/-}$ and *Vrk2*$^{-/-}$ mice. A Open field test. No significant difference was seen in the total distance (*Vrk2*$^{+/+}$ vs. *Vrk2*$^{+/-}$, *P* = 0.133; *Vrk2*$^{+/+}$ vs. *Vrk2*$^{-/-}$, *P* = 0.173) and central exploration time between different groups (*Vrk2*$^{+/+}$ vs. *Vrk2*$^{+/-}$, *P* = 0.981; *Vrk2*$^{+/+}$ vs. *Vrk2*$^{-/-}$, *P* = 0.218). *Vrk2*$^{+/+}$, *n* = 6; *Vrk2*$^{+/-}$, *n* = 10; *Vrk2*$^{-/-}$, *n* = 9. **B Elevated plus maze.** No significant difference was found in distance ratio (*Vrk2*$^{+/+}$ vs. *Vrk2*$^{+/-}$, *P* = 0.771; *Vrk2*$^{+/+}$ vs. *Vrk2*$^{-/-}$, *P* = 0.666) and time ratio (*Vrk2*$^{+/+}$ vs. *Vrk2*$^{+/-}$, *P* = 0.644; *Vrk2*$^{+/+}$ vs. *Vrk2*$^{-/-}$, *P* = 0.987) into the open arms between different groups. *Vrk2*$^{+/+}$, *n* = 6; *Vrk2*$^{+/-}$, *n* = 10; *Vrk2*$^{-/-}$, *n* = 9. **C Sucrose preference test.** *Vrk2*$^{+/+}$ vs. *Vrk2*$^{+/-}$, *P* = 0.445; *Vrk2*$^{+/+}$ vs. *Vrk2*$^{-/-}$, *P* = 0.279. *Vrk2*$^{+/+}$, *n* = 6; *Vrk2*$^{+/-}$, *n* = 6; *Vrk2*$^{-/-}$, *n* = 9. **D Tail suspension test.** Immobility time: *Vrk2*$^{+/+}$ vs. *Vrk2*$^{+/-}$, *P* = 0.854; *Vrk2*$^{+/+}$ vs. *Vrk2*$^{-/-}$, *P* = 0.994. Curling time: *Vrk2*$^{+/+}$ vs. *Vrk2*$^{+/-}$, *P* = 0.9771; *Vrk2*$^{+/+}$ vs. *Vrk2*$^{-/-}$, *P* = 0.0927. *Vrk2*$^{+/+}$, *n* = 6; *Vrk2*$^{+/-}$, *n* = 10; *Vrk2*$^{-/-}$, *n* = 9. $^{\#}P < 0.1$, One-way ANOVA for A-D. Error bars represent mean ± standard deviation (SD).

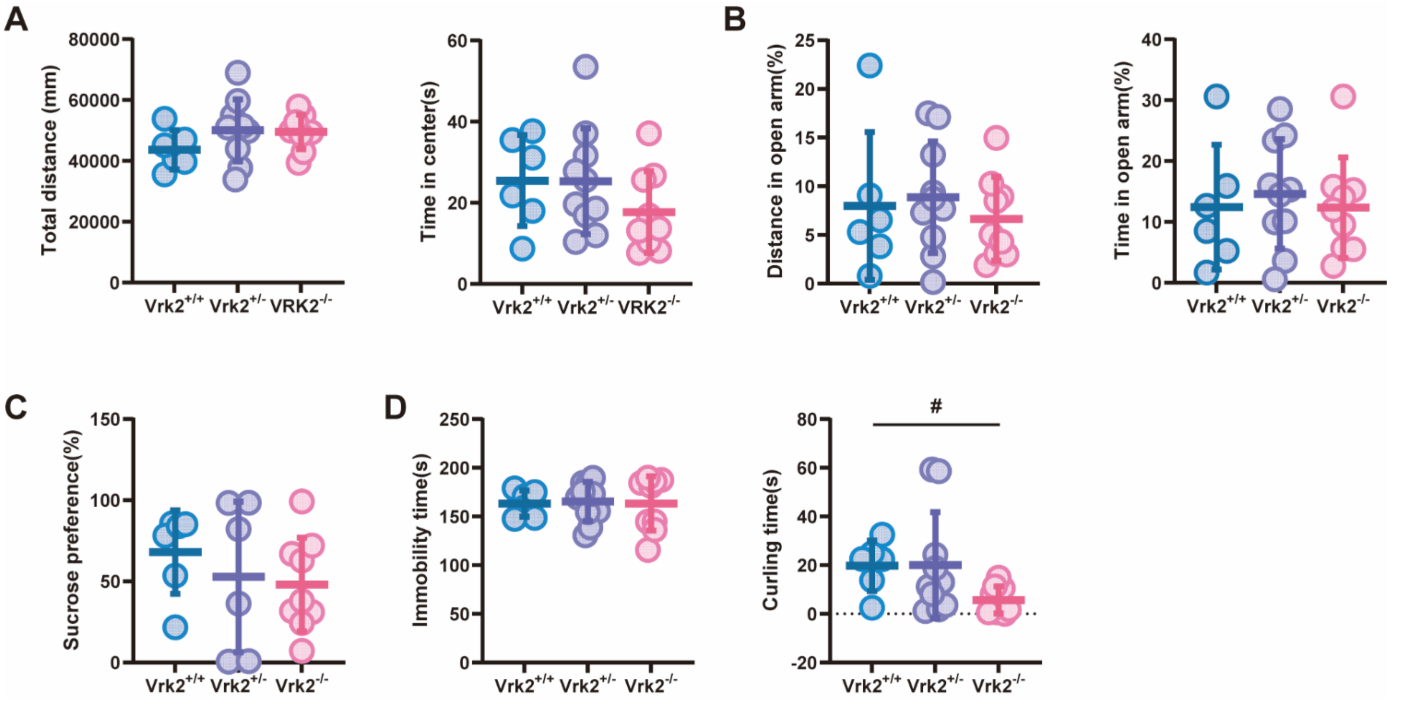

References

1. Genomes Project Consortium, Auton A, Brooks LD, Durbin RM, Garrison EP, Kang HM, Korbel JO, Marchini JL, McCarthy S, McVean GA *et al*: **A global reference for human genetic variation**. *Nature* 2015, **526**(7571):68-74.
2. Barrett JC, Fry B, Maller J, Daly MJ: **Haploview: analysis and visualization of LD and haplotype maps**. *Bioinformatics* 2005, **21**(2):263-265.
3. Ward LD, Kellis M: **HaploReg: a resource for exploring chromatin states, conservation, and regulatory motif alterations within sets of genetically linked variants**. *Nucleic Acids Res* 2012, **40**(Database issue):D930-934.
4. Ritchie GR, Dunham I, Zeggini E, Flicek P: **Functional annotation of noncoding sequence variants**. *Nat Methods* 2014, **11**(3):294-296.
5. Fromer M, Roussos P, Sieberts SK, Johnson JS, Kavanagh DH, Perumal TM, Ruderfer DM, Oh EC, Topol A, Shah HR *et al*: **Gene expression elucidates functional impact of polygenic risk for schizophrenia**. *Nat Neurosci* 2016, **19**(11):1442-1453.
6. Ai H, Shi XF, Hu XP, Fang WQ, Zhang B, Lu W: **Acute stress regulates phosphorylation of N-methyl-d-aspartate receptor GluN2B at S1284 in hippocampus**. *Neuroscience* 2017, **351**:24-35.
